# A Sophisticated Onscreen Smart Framework for Predicting Diabetes in Remote Healthcare

**DOI:** 10.3390/diagnostics16040532

**Published:** 2026-02-11

**Authors:** Koteeswaran Seerangan, Premalatha Gunasekaran, Nithya Rekha Sivakumar, Resmi Ravi Nair, Malarvizhi Nandagopal, Neeba Eralil Abi, Nalini Manogaran

**Affiliations:** 1 Department of Computer Science and Engineering, R.M.K. Engineering College (Autonomous), Chennai 601206, Tamil Nadu, India; s.koteeswaran@gmail.com; 2 Department of Data Science and Business Systems, School of Computing, Faculty of Engineering and Technology, SRM Institute of Science and Technology, Kattankulathur, Chennai 603203, Tamil Nadu, India; premalag@srmist.edu.in; 3 Department of Computer Sciences, College of Computer and Information Sciences, Princess Nourah bint Abdulrahman University (PNU), P.O. Box 84428, Riyadh 11671, Saudi Arabia; nrraveendiran@pnu.edu.sa; 4 Department of Computer Science and Engineering, Saveetha Engineering College (Autonomous), Chennai 602105, Tamil Nadu, India; resmi.gie@gmail.com; 5 Department of Computer Science and Engineering, Vel Tech Rangarajan Dr. Sagunthala R&D Institute of Science and Technology, Avadi, Chennai 600062, Tamil Nadu, India; drnmalarvizhi@veltech.edu.in; 6 Department of Computer Science and Engineering, Alliance University, Bengaluru 562106, Karnataka, India; neeba.ea@alliance.edu.in; 7 Department of Computer Science and Engineering (Cyber Security), S.A. Engineering College (Autonomous), Chennai 600077, Tamil Nadu, India

**Keywords:** remote healthcare, diabetes prediction, Deep Learning (DL), classification, Artificial Intelligence (AI), Brass Optimized Learning-based Diabetes Prediction (BOLD), Deer Hunting Optimization (DHO), Brassy Pelican Optimization (BPO), Recurrent Neural Network—Long Short Term Memory (LSTM)

## Abstract

**Background/Objectives:** Diabetes is one of the most familiar and common diseases among people currently, and is a type of metabolic disease that is caused due to high levels of sugar in the blood for longer periods of time. If the disease is predicted at an earlier stage, the severity and risks associated with diabetes are significantly reduced, which helps to save the lifespan of people. In earlier investigations, various kinds of automated models based on artificial intelligence (AI) were developed for this purpose. However, key issues still revolve around the lack of robustness, dependability, and precise prediction. The motivation behind the proposed study is to design and develop an automated tool for the diagnosis of chronic disease with the use of novel AI methodology. **Methods:** For this purpose, a new detection framework is introduced, known as the Brass Optimized Learning-Based Diabetes Prediction (BOLD) model for remote healthcare applications. By using this kind of optimization-integrated deep learning technique, the overall performance and efficiency of the diabetes detection system are maximized. This framework preprocesses the input diabetes dataset before performing the data splitting, normalization, and cleaning activities. Next, the best attributes for improving the prognostic performance of the classifier are chosen using the Brassy Pelican Optimization (BPO) procedure. The Hunting Optimized Recurrent Neural Network—Long Short-Term Memory (RNN-LSTM) method is used to categorize the people into those who are diabetic and those who are not based on the chosen attributes. The approach employs a Deer Hunting Optimization (DHO) method to choose the hyperparameters needed to make an informed choice. A variety of parameters have been employed to confirm the results, which are evaluated for performance verification using the PIDD, Indonesia diabetic database, and kidney disease dataset. **Results:** The BOLD framework is successful to the extent that it has been able to achieve several metrics of comparably good results, such as an RMSE value of 0.015, a Cohen’s Kappa measure of 0.99, a precision of 0.991, a recall of 0.99, an accuracy equal to 0.996, and an AUC equal to 0.99. **Conclusions:** It is also remarkable that a very short time of 0.8 s was enough for it to deliver this kind of performance, making it a neat combination of both time and power efficiency.

## 1. Introduction

**Figure 1:**
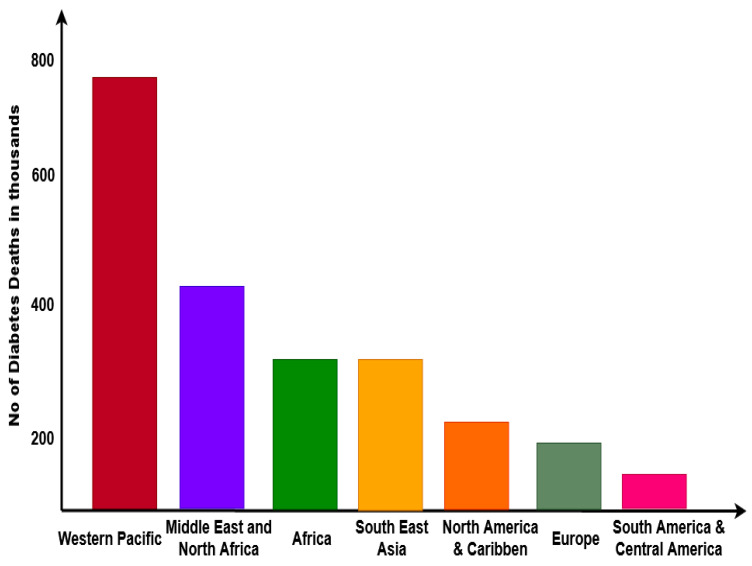
Recent statistics on diabetes death rate.

In the healthcare community, diabetes is considered the most dangerous hazard to people around the world, and its growth has gradually increased each year [1,2]. According to medical statistical reports provided by the International Diabetes Foundation (IDF), it is estimated that approximately 385 million people are affected by diabetes around the world. Also, its impact has greatly increased in the past few decades, hence constituting a global threat [3,4,5]. Currently, diabetes is the most prevalent cause of death, and it has remained prevalent for some time. Moreover, it has increased to 48%, and 630 million people have been affected by this dangerous disease in 2020. Nevertheless, diabetes is controllable and can be prevented by making lifestyle changes. Additionally, these modifications can lessen the risk of coronary artery disease and cancer [6]. Consequently, there is an urgent need for a forecasting tool that may assist doctors in early disease diagnosis and, as a result, put forward the lifestyle modifications needed to avert the fatal disease’s progression. The recent statistical model of diabetes is graphically illustrated in Figure 1, where the number of deaths around the world before the age of 60 in recent years is illustrated.

The World Health Organization (WHO) reports that diabetes is the 7th major reason for the death rate [5,7], which is the cause of heart disease, stroke, and kidney failure problems. Therefore, it needs to be caught early to give patients the right care and keep them from developing serious health issues. The physician’s expertise and knowledge are used to forecast and diagnose the disease during the initial treatment, although this analysis might be vulnerable to error. The healthcare industry gathers a lot of data, but it cannot use those data to see developments that are not obvious to make good judgments. Manual judgments can be extremely risky for the early detection of disease, since they rely on a medical professional’s findings and assessment, which may not be consistently reliable [2,8,9]. There may be certain hidden patterns that have effects on perceptions and results. Patients are consequently receiving subpar care, necessitating the development of a sophisticated mechanism for the prompt identification of disease with computerized diagnosis and a higher precision. Different data mining and learning algorithms can produce effective results with reliable precision based on a variety of undetected flaws and hidden patterns [10].

Diabetes has become one of the biggest global health challenges the world faces today; as per the latest figures from the International Diabetes Federation, the adult population living with diabetes is currently 537 million people globally. It is estimated that this figure will reach 643 million by 2030 and 783 million by 2045. Besides being a common disease, diabetes is quite deadly, as it eventually causes the death of 6.7 million people each year. Of the total cases of diabetes, almost half remain undiagnosed, hence the development of severe complications, cardiovascular disorders, renal damage, vision loss, and neuropathy without the patient being aware. The disease’s economic impact is comparably scary. The total global healthcare expenses due to diabetes have eclipsed 966 billion US dollars, which means that there has been a considerable increase over the last ten years. These statistics point to the absolute necessity for an early, accurate diagnostic method that can be easily deployed, which would indeed be a preventive healthcare revolution in rural areas and resource-poor regions. Early diagnosis remains the most effective way of cutting down the death rate, mitigating the money issue, and enhancing patients’ quality of life; thus, it is the main reason behind the rise in advanced and reliable AI-based models for prediction. Most of the studies on automatic diabetes identification by means of machine learning and deep learning have been comprehensively reviewed. However, to a large extent, the issues of these models regarding accuracy, robustness, and generalizability have not been resolved; therefore, their potential is limited.

A significant number of traditional methods are very dependent on the limited feature representations or are developed with poorly optimized learning architectures that are incapable of understanding the complex non-linear patterns in biomedical data. The prediction results of these methods are incorrect when they are used to analyze noisy, imbalanced, or heterogeneous data. Moreover, they usually have a decreased stability and produce different outcomes for various populations. In addition to that, a great number of prior works have been deficient in performance-efficient feature selection, and the writers of these works have used fixed or manually set hyperparameters; consequently, the models are vulnerable to overfitting and have low levels of adaptability in real healthcare scenarios. The obstacles hereby reveal the necessity for a superior system that is capable of integrating optimization-centered learning to upgrade feature discrimination, stabilize predictions, and enhance overall diagnostic reliability at a higher level, thus the emergence of BOLD (Brass Optimized Learning-Based Diabetes Prediction) as a technology for the remote, strengthened, and data-driven healthcare support sector.

Diabetes is the main reason for a large part of social and economic costs, primarily due to the length of the disease and the seriousness of the complications that follow it, which additionally include diabetic kidney disease, retinopathy, neuropathy, cardiovascular disorders, as well as the condition of the patient becoming worse if infected. In addition to these complications, which lead to a decrease in quality of life and raise the mortality rate, diabetes patients also, through a long treatment process, frequently visit hospitals and deal with productivity loss, which are heavy financial burdens on healthcare systems and families. Therefore, both early and accurate diabetes detection is required, as a timely diagnosis allows for the implementation of management strategies which delay or prevent the development of severe complications. Consequently, by identifying the most vulnerable individuals before significant organ damage occurs, automated detection systems like the BOLD framework can remove the heavy load caused by diabetes on both individuals and society, thus making patient outcomes better and healthcare costs lower. This mutual reliance shows the significance of the building of strong, accurate, and efficient predictive models, as these, being direct, are the ones that have the greatest impact on the reduction in the social and economic consequences of diabetes and therefore the related complications.

Numerous types of methods based on data mining have been developed for extracting hidden trends from vast amounts of healthcare data in response to the daily rising cost of diabetes. Additionally, such information can be used for automated diabetes prediction and decision making. When one takes into account the limits of present methods in resolving security concerns in SDN and the precise prediction of diabetes, the research gap in the literature becomes evident. While some research just addresses diabetes detection or SDN security, few studies combine the two topics into a single, cohesive framework. Moreover, current methods frequently lack accuracy, resilience, and efficiency, especially when dealing with big datasets and dynamic network environments. By providing a novel method that simultaneously increases diabetes prediction accuracy and security in SDN-IoT contexts, the suggested BOLD model fills this gap. BOLD seeks to address the limitations of current approaches by utilizing deep learning algorithms and advanced optimization techniques, offering a thorough and effective solution for addressing these two difficulties in healthcare.

The BOLD framework is a major step ahead in methodology that amalgamates the optimization-driven feature selection, the deep learning-based classification and the adaptive hyperparameter tuning, all three, into a single robust model. This is not only a mere recasting of the prior concepts with different names, but the framework also goes beyond this to bring in the new-age concepts for the chronic disease prediction-specific issues. Brassy Pelican Optimization (BPO) is one of the new methods that have been introduced to facilitate the identification of the most informative features of the complex, high-dimensional datasets transparently and systematically. In contrast with traditional selection methods, BPO promulgates an innovative pelican-metaheuristic foraging behavior. Therefore, integrating it into the BOLD pipeline shows that the diagnostic system is operating at the most discriminative features and, hence, it can accurately perform its prognostic function. Additionally, Deer Hunting Optimization (DHO) is yet another alternative that was given to resolve the hyperparameter problem of the Recurrent Neural Network—Long Short-Term Memory (RNN-LSTM) classifier, just like BPO.

In the same way as genetic algorithms or particle swarm, conventional optimization methods experience the issues of premature convergence or slow adaptation of high-dimensional parameter space. On the premise of cooperative hunting, DHO not only encourages a fast and accurate search of the hyperparameter landscape but at the same time it allows a silent performance along with faster converging. The integration of BOLD and DHO enables the fine-tuning of RNN-LSTM to be so good that it obtains the maximum classification accuracy and consequently, predictions become more trustworthy. The BOLD model has to be considered a genuine methodological contribution that is not even slightly close to other healthcare problem frameworks because it is distinguished by its robustness, preciseness, and adaptability when the characteristics of BPO and DHO have been smartly used for hyperparameter tuning.

There have been numerous attempts to conduct research in this area to predict diabetes using Artificial Intelligence (AI) techniques [11,12,13]. Deep learning techniques are more appropriate for predicting diabetes when compared to machine learning techniques. However, it has issues with a complex system, longer processing times, and imprecise disease identification [14,15]. The proposed attempt intends to construct a cutting-edge and potent deep learning architecture model for diabetes prediction, which has the following objectives:

- The data cleaning, attribute normalization, and splitting processes are carried out in the course of preprocessing, which follows the acquisition of the dataset.
- The Brassy Pelican Optimization (BPO) algorithm is used to parse the preprocessed dataset and extract the most relevant features, hence reducing the dimensionality of features for better classification.
- The patients with and without diabetes are divided using the Hunting Optimized Recurrent Neural Network—Long Short Term Memory (HO-RNN-LSTM) technique based on the selected attributes.
- During classification, the Deer Hunting Optimization (DHO) technique is utilized to fine-tune the hyper parameters for effective decision making.
- In addition, a PIDD dataset, Indonesia diabetic database and kidney disease dataset available from the UCI repository are used in this study for performance assessment, where a variety of parameters are used for the evaluation of results.

In essence, the primary aim of this research work was to develop and actually implement a system that is characterized mainly by the features of being super robust and precise and having the capability of early automatic detection along with rightful classification of diabetes and other related chronic diseases by using complex artificial intelligence methods. Generally, early detection of diabetes is a matter of great importance, particularly if we consider the reduction in the rate of the disease, its side effects, and even the shortening of the life span to some extent. In addition, the automated models that are here to help have minor problems, such as the lack of precision and the robustness of the models. A very promising algorithm for solving these kinds of problems is the BOLD framework, which depends heavily on Brass Optimized Learning (BOLD). This model basically takes feature selection through deep learning classification and uses hyperparameter tuning as its main operation to get high predictive accuracy and stability in remote healthcare scenarios. As a clinical instrument, this model has the potential to make the process of decision making easier and, consequently, health care management will be simpler. Besides that, the aspect of the combination of preprocessing, feature selection, classifications, and optimization being an all-in solution should also be considered. Basically, this research is associated with four major objectives. The first goal, according to the study, is about the handling and organizing of diabetes data. The work consists of normalization, cleansing, and partitioning into training, validation, and test sets, with the aim of ensuring high quality of data used for model development. The second goal is about creating and executing Brassy Pelican Optimization (BPO) algorithm for feature selection. Improvement of the system in diagnostic performance will be achieved by the implementation of this algorithm through the selection of those features that are at once informative and discriminative. The third goal is the implantation of Deer Hunting Optimization (DHO) for the purpose of attaining hyperparameters in the RNN-LSTM classifier which is the one responsible for optimum learning and predictive results.

The following units make up the remaining sections of this paper: The thorough literature evaluation for machine learning and deep learning strategies for diabetes categorization and prediction is included in Section 2. The suggested diabetes detection framework is explained in detail in Section 3, together with the required algorithms and descriptions. The performance and effectiveness of the suggested system are validated in Section 4 using the widely used benchmarking datasets and assessment metrics. The results and future work are presented in Section 5, which also serves as the paper’s conclusion.

## 2. Related Works

This section looks into various conventional methods that have been used to predict diabetes based on the available information. Additionally, it looks into each model’s benefits and drawbacks in relation to detection effectiveness and enhanced performance results.

Hasan, et al. [16] used a combination of machine learning classification methods to predict diabetes. For correct classification training and testing procedures, many characteristics like age, body mass index, tricep strength, blood pressure, glucose level, and pregnancy status have been taken into consideration in this study. This system first performs data preprocessing for outlier rejection and missing value eradication. The z-score normalization procedure is used to perform data imputation in this case, which helps to standardize the dataset for improved categorization. The data is then trained for prediction using K-fold cross-validation while maintaining the samples at each stage. Additionally, the grid searching method is used for hyperparameter tweaking, which increases the accuracy of prediction. Jaiswal, et al. [17] examined the most recent developments in diabetes prediction machine learning methods. The primary goal of this investigation is to examine the intriguing patterns that are used to predict diabetes. The most popular machine learning methods, including Artificial Neural Networks (ANN), Support Vector Machines (SVM), Bayesian Networks (BN), Random Forests (RF), and many others, are covered in this review. Additionally, this study has combined other datasets, including PIMA, digestive and kidney disease, national health guard affairs, and others. Mujumdar, et al. [18] established a big data analytical system based on patient data such as age, blood pressure, obesity, and other factors to identify diabetes mellitus. In this instance, the k-means clustering algorithm is used to classify the patients as diabetic or non-diabetic based on feature properties. The main shortcomings of the proposed framework, however, are its poor prediction effectiveness and limited detection effectiveness.

Ahmed, et al. [19] designed a conceptual framework based on fused machine learning to predict diabetes. Here, the performance and outcomes of the given framework have been validated using measures like accuracy and miss rate. Islam, et al. [20] implemented a likelihood-based prediction model for the detection of diabetes from the patients’ medical data. In this study, decision making has been done using four different categorization systems, including NB, RF, LR, and J48. For the purpose of making informed decisions, additional characteristics including polyuria, polydipsia, itching, irritation, and alopecia, are taken into account. In this research, performance is evaluated based on the weighted average value of these categorization techniques. Thakkar, et al. [21] employed a fuzzy logic technique for the prognosis of diabetes from the patients’ medical data. In this study, some of the different datasets such as LARS, Pima, Abel vikas, and diabetes risk are used for system deployment and validation. Moreover, the authors aim to minimize the time complexity and enhance the classification accuracy by analyzing the specific features from the given data.

Selvachandra, et al. [22] delivered a thorough study of the most recent state-of-the-art techniques applied in the creation of a computationally assisted diabetes screening tool. SVM, CNN, ANN, and other modern approaches have been used in this work to conduct a thorough performance analysis and investigation. Furthermore, the study showed that when compared to alternative learning approaches, CNN-based deep learning techniques offer better accuracy. Uddin, et al. [23] intended to predict type-2 diabetes with the use of machine learning approaches. In addition, they have applied SMOTE and oversampling approaches for an efficient diabetes detection and type classification. According to the ranked features and level of efficiency, the effectiveness of machine learning methodologies is determined in this study. Thotad, et al. [24] used a deep learning architecture model based on EfficientNet to improve the MCC, accuracy, precision, and recall metrics. Furthermore, the authors focused on creating a straightforward and inexpensive diabetic diagnostic instrument. Vij, et al. [25] implemented a refined RF classification strategy to identify diabetes early on. Here, the results of the provided algorithm have been assessed and evaluated using the top 5-fold cross validation. Furthermore, this model’s main advantages are its great efficiency and improved interpretability.

Kazerouni, et al. [26] validated and compared the performance of four different classification approaches for the prognosis of type 2 diabetes, which includes the models of SVM, ANN, LR, and KNN. Here, it is mentioned that the accuracy of the single classification technique may not be effective; hence, the authors used a combination of four different techniques for an accurate prediction. Shuja, et al. [27] performed a SMOTE analysis to predict diabetes from the imbalanced data with high accuracy. Typically, the timely prediction and prognosis could be more useful to maximize the lifetime of the people affected by diabetes. Moreover, exploring some of the unknown patterns from the medical data could improve the decision-making performance of the diagnosis system in healthcare applications. Raja, et al. [28] deployed a hybridized Fuzzy C-Mean (FCM) integrated Particle Swarm Optimization (PSO) algorithm for the prognosis of diabetes from the medical data. The purpose of this work is to implement a clustering-based prediction with negative outcomes. Hassan, et al. [29] utilized a variety of classification techniques such as DT, SVM, and NB for the prediction of diabetes mellitus. The examination revealed that the PIMA dataset was used by the vast majority of previous studies for system validation and evaluation. Rastogi, et al. [30] deployed a new robust framework based on Deep Neural Network (DNN) for the detection of diabetes. This study mainly focused on handling unstructured medical records with the use of mining techniques including preprocessing and classification. In this strategy, the Restricted Boltzmann Machine (RBM) is also applied to perform pre-training before feature extraction and classification. Ram, et al. [31] applied a Guided Neural Network (GNN) to predict the existence of diabetes with the use of patients’ previous medical history. In this model, the error value is computed for the training data at each iteration for maximizing the level of detection accuracy. Almazroi, et al. [32] developed a clinical decision support system for accurately predicting cardiac disease with the use of a deep learning-based dense neural network approach. Moreover, this study investigated the merits and demerits of some recent classification methods used for detecting heart disease. Table 1 presents the comprehensive survey to examine several machine and deep learning techniques used for diabetes prediction.

## 3. Proposed Methodology

**Figure 2:**
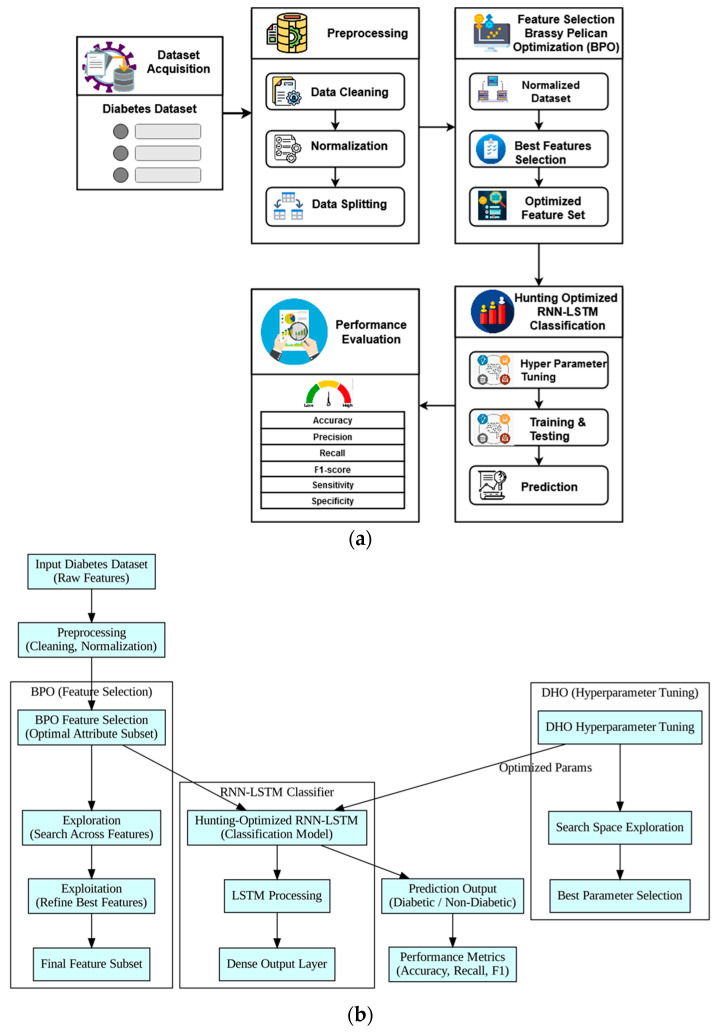
(**a**) Overview of BOLD framework. (**b**) Flow of BOLD framework.

The proposed framework for the prognosis of diabetes is detailed in this section, along with a flow diagram and explanations. The main contribution of this work is the development of a concise and effective framework for remote healthcare applications known as Brass Optimized Learning based Diabetes Prediction Model (BOLD). In this work, a collection of intelligent algorithms is put to use to precisely diagnose diabetes from the medical history of the patients. The suggested BOLD system’s flow model is depicted in Figure 2, where the following processes are carried out:

- Medical data acquisition;
- Data cleaning and preprocessing;
- Brassy Pelican Optimization (BPO) for feature dimensionality reduction;
- Hunting Optimized Recurrent Neural Network—Long Short Term Memory (RNN-LSTM) for disease prediction;
- Deer Hunting Optimization (DHO) for parameter tuning;
- Performance assessment.

The overall block diagram and flow diagram of the proposed diabetes detection system using BOLD model are represented in Figure 2a,b. The Pima Indian Diabetes Dataset (PIDD), Indonesia diabetic database, and kidney disease dataset are well-known and widely used diabetes datasets, and they are used as the input data for processing. After preprocessing the input dataset, multiple procedures including cleaning, normalization, and separating the training and testing set are carried out [38]. The optimal features are then selected and the dimensionality of the preprocessed data is decreased using the cutting-edge BPO approach. The HO-RNN-LSTM is used to identify individuals with diabetes and those without it with accuracy by utilizing the selected training characteristics. The RNN-LSTM hyperparameters are adjusted during this process using the Deer Hunting Optimization (DHO) algorithm, which helps the classifier make correct decisions with minimal computing overhead. The purpose of the performance evaluation is to validate and compare the results of the suggested BOLD framework. The ability of the proposed BOLD framework to accurately forecast diabetes using the best-fit features extracted from the medical dataset is its main contribution. Previous studies have found a multitude of ML- and DL-related pathways for diabetes type classification and prediction.

The major single feature that differentiates the Brassy Pelican Optimization (BPO) algorithm from other cell-inspired algorithms is its search strategy, which is not only biophysically more realistic, but also is by nature more efficient in obtaining a balance between exploration and exploitation than those which are merely heuristic-based. In BPO, feature selection is performed by combining BPO with PSO, while GA is employed for hyper-parameter tuning of the XGBoost classifier in the BOLD model. In one way or another, authors by developing BPO have confronted the problems of premature convergence and getting stuck at a local minimum in meta-heuristics. To make a long story short, BPO is based on the idea of pelicans doing things such as flock formation, diving, hunting, and repositioning through different behavioral stages, which eventually enable the algorithm to be the closest one to the continuous multi-dimensional space in an approximate way. For example, the main idea of BPO is that pelicans change their search paths as they receive signals from the environment, local areas, and the whole pelican community; thus, they can localize optima on a global level. Since the model is a biological one, this highly adaptable model not only is capable of selecting the most informative and discriminative features from high-dimensional diabetes datasets, but it can also remove noise and redundancy, thereby making the classifier more efficient in learning. Therefore, BOLD, whose central part is such a behavior-based optimizer, is firm evidence that the chosen feature subset is a means of maximizing predictive quality, enhancing feature separability, and broadening the overall decision-making capacity of the downstream deep learning model.

Deer Hunting Optimization (DHO) is another novel hyperparameter-tuning tool that derives its idea from the systematic hunting strategies of deer predators, and thereby it differs from the usual gradient-based or random-search optimization methods just by its source of inspiration. This feature endows the DHO with the ability to control the degree of its movements and thus, it may not come across the problem of premature convergence and at the same time, it may also have the ability to accommodate the current training process of the RNN-LSTM model. Therefore, the algorithm is the only one that deals with the most important parameter optimization problem, like learning rate, number of hidden units, dropout rate, and recurrent depth; thus, stable convergence and improved prediction accuracy are the results. Consequently, the BOLD model empowered by these two pioneering optimization methods can, therefore, be a step ahead of the traditional ones. Such a capability finds its manifestation in the model’s durability, flexibility, and enhanced predictive accuracy.

Conventional approaches have certain drawbacks, too, such as laborious implementation procedures, lower effectiveness, protracted training and testing periods, and computing complexity. Thus, the BOLD framework—which was developed to yield high-performing outcomes in the diagnosis and type categorization of diabetes—is employed in the proposed study. Given that the recommended strategy makes use of state-of-the-art mining techniques that are perfect for applications involving remote healthcare, most contemporary remote healthcare apps use artificial intelligence (AI) algorithms and other mining techniques to remotely analyze patient medical data. Healthcare professionals are relieved of this duty when diseases can be diagnosed automatically and with high accuracy thanks to HO-RNN-LSTM. The new algorithm for diabetes prediction known as HO-RNN-LSTM was created by fusing the LSTM-RNN model with the hunting optimization technique. Here, the disease is predicted using the available data, and the activation function is approximated using HO. Furthermore, because it accurately diagnoses the condition with better detection outcomes, the BPO and HO-RNN-LSTM combo is novel to this sector for diabetes prediction. The patients’ medical information can be followed and evaluated utilizing the digitalized sensors, using BOLD for disease prediction. As a result, BOLD is ideal for applications involving distant medical care. The overall time complexity for the proposed work is $O(n\times m^{2})$ .

Even though numerous hybrid-methodology-based works have been realized to facilitate the integration of optimization algorithms with machine learning or deep learning techniques for diabetes prediction, the BOLD framework still looks like it is very far away from these and keeps a large difference in many aspects. The majority of present models are merely feature selection-oriented or they treat the tuning of hyperparameters as two separate tasks with no overlap, and they predominantly rely on traditional optimization methods such as Particle Swarm Optimization (PSO), Genetic Algorithm (GA), or gradient-based methods for solving optimization problems. On the other hand, the BOLD framework amalgamates two innovative metaheuristic optimization methods, namely Brassy Pelican Optimization (BPO) for feature selection and Deer Hunting Optimization (DHO) for hyperparameter tuning, into one seamless flow.

The dual-optimization strategy not only spots the most informative features but also, by the RNN-LSTM classifier, achieves machine conversion to the highest performance, thus a very rare combination in prior studies, which are only partial in terms of comprehensiveness. Moreover, this composition also very much focuses on efficiency and quick calculation, which results in a low system execution time, a very important feature for the real-time remote healthcare application. Another major difference, which is just as important, is the very detailed validation and assessment process used. The BOLD framework is beyond just the accuracy or F1 score that are the only things usually presented by previous hybrid models. The BOLD framework not only adopts a multi-metric evaluation that includes RMSE, Cohen’s Kappa, precision, recall, accuracy, AUC, and computational time, along with the use of k-fold cross-validation to test the generalization and robustness of results, but it also goes further. Besides that, the combination of both structured diabetes and kidney disease datasets not only allows the model to have a broader range of diseases to handle but also implies the model’s non-standardness for diabetes prediction tasks. As such, it becomes a new contribution to the field of automated chronic disease detection.

The BOLD framework, which has been proposed, brings with it a great deal of novelty and real-world application value that is not present in the existing diabetes prediction models found in the literature. One of the main distinctions between BOLD and traditional methods is that the choice of machine learning classifiers and single-stage optimization in conventional models is the main way; BOLD goes on to fuse Brassy Pelican Optimization (BPO) for feature selection and Deer Hunting Optimization (DHO) for hyperparameter tuning of the RNN-LSTM classifier, resulting in the dual-optimization technique. Consequently, it not only selects features that are generatively and statistically significant, but at the same time, the deep-learning architecture is optimally set up so that predictive accuracy and stability improve.

Moreover, whereas most other models only consider magnitudes of accuracy as their KPI, BOLD focuses on a more encompassing one with the presence of precision, recall, F1-score, AUC, RMSE, Cohen’s Kappa, and computational efficiency as a part of the evaluation criteria, hence offering better insight into the performance by the models. The layout of the new product is also enabling. Besides that, it is suitable for remote health care that requires quick real-life monitoring, and early intervention is therefore a hot area for research. It is the BOLD framework pulling all the problems in previous works such as overfitting, limited generalizability, and lack of robustness across diverse datasets, thus making it a true methodological step forward, a dependable as well as a scalable approach, and, last but not least, a solution relevant for clinical practice in early diabetes detection, and consequently, alleviation of the associated complications.

The central theoretical move of the BOLD framework is its dual-layer optimization–deep learning architecture, which in essence signifies a dual-layer optimization–deep learning architecture. That theoretical move is its dual-layer optimization–deep learning architecture. That theoretical move is its dual-layer optimization–deep learning architecture. That theoretical move is its dual-layer optimization-driven deep learning design that interacts with the existing problem of the trade-off between accuracy and adaptability in AI-based medical diagnosis systems in such a way that the problem gets solved. To be more exact, the BOLD approach goes beyond the usual fixed structure machine learning algorithms or simple neural networks. Instead, it employs a layered and interlinked optimization and deep-learning strategy in such a way that feature selection and hyperparameter tuning are simultaneously performed in an auto-regulated manner.

The first part of the method is basically the invention of the Brassy Pelican Optimization (BPO) algorithm, a metaheuristic that imitates pelican foraging and socialization behaviors in nature to dynamically select the most discriminant and information-rich features of the input data. Also, the BPO finds those features that have the greatest discriminatory power and information content in the dataset by simulating pelican foraging and socializing behaviors. By the way, the features will be less redundant and noisy, and therefore the engine will focus on the core metabolic indices that are most correlated with diabetic risk. Up to this point, the method has not only extended the model’s capability of generalization across different medical datasets but has also drastically lowered the computational power requirements of AI frameworks which have been their bottleneck.

Moreover, the BOLD structure is the reason for a major theoretical breakthrough, the Deer Hunting Optimization (DHO) algorithm for hyper-parametric fine-tuning of the hunting-optimal RNN–LSTM classifier that uses the DHO algorithm. The authors of the paper argue that the inhibitory landscapes are non-convex in normal LSTM networks, and thus the sensitivities to parameter choice and initialization that lead to the training processes are difficult. To handle this problem, the authors have used the DHO method. The DHO method imitates the collaborative hunting techniques among deer groups, so by its use in the framework, it actually results in learning parameters (such as learning rate, number of memory units, and dropout ratios) reaching global optimum points instead of local minima. The time-related enhancements of the LSTM due to this optimization also suggest that the technique is now more capable of following blood glucose changes and other metabolic dependencies, thus unveiling deeper non-linear relations, which models like Random Forest, SVM, or shallow ANNs would scarcely find or would simply overlook.

Brassy Pelican Optimization (BPO) algorithm represents the behavior of pelicans when they are foraging as a group. Particularly, the model depicts their coordinated diving and the cooperative localization of prey to get the food. As a result, this essentially gives rise to a dual-mode search mechanism—global exploration is carried out by random trajectory updates that emulate flock movement over large search areas, while local exploitation is executed through the adaptive velocity correction at prey capture. In addition, an inertia weight component is used to allow a more gradual convergence and also to decrease the probability of premature stagnation, which happens most of the time in typical algorithms like Particle Swarm Optimization (PSO) or Genetic Algorithms (GA).

Likewise, the Deer Hunting Optimization (DHO) algorithm can be referred to as the model of the evolutionary cooperational strategy of the deer, where the animals demonstrate both the intelligence of an individual (searching for food patches) and the coordination of a group (encircling prey). The combined behavior, in mathematical terms, amounts to two control parameters—group attraction coefficient and leader-driven pursuit coefficient—that change their values depending on fitness variance, thus ensuring that the proper balance between intensification and diversification is maintained at all times. DHO’s adaptive movement rules make it possible for the RNN–LSTM network to efficiently travel through complex non-convex error landscapes; hence, the network is not vulnerable to the problem of vanishing gradients and local minima. At a collective level, the theoretical underpinning of BPO and DHO is not only a natural analog of self-organizing optimization processes but also it endows them with stable convergence properties, diversity preservation, and computational scalability; thus, both algorithms are suitable for medical prediction tasks in the real world where data heterogeneity and nonlinearity are the major challenges.

### 3.1. Preprocessing

Data preprocessing is the procedure used to improve the viability of real-world data for the process of mining. The raw data collected from the real-world environment are larger in size, highly erratic and riddled with missing values and unclear information. When mining or modelling results are obtained, all of these factors lead to a decrease in the overall quality of the data. Hence, data enhancement methods known as data prior treatment must be applied before processing or analyzing the data. Such a process can be carried out using a variety of approaches to get the data ready for analysis. The goal of preprocessing data is to convert the unprocessed information into an organized form that will be simpler and more useful for subsequent processing stages. The min-max approach is used in the beginning in order to normalize the data. Since all training data are on the same scale, for instance between 0 and 1, the normalization could be more useful to reduce the training time.

The process of transforming the value of an infinite data attribute into a sequence of finite intervals by avoiding the information loss in the data is known as data separation. In the proposed system, the data cleaning and normalization operations are carried out at the earlier stage for data quality improvement. During this process, the unwanted attributes, missing fields or information, and redundant attributes are identified, and removed with the use of standard min-max normalization approach. Typically, the min-max is one of the most well-known preprocessing algorithms, specifically applied in many existing applications for the purpose of data preprocessing and normalization. In this technique, the distance between the attributes is estimated for normalizing the data, since the raw dataset comprises the noisy information, which may degrade the performance of the classifier with high training and testing time. Also, it reduces the speed of classification with increased data dimension. Hence, the preprocessing is done at the beginning stage for improving the quality of input dataset used for feature optimization and classification.

The BOLD framework is able to reach its comprehensive and well-structured workflow through a smartly designed architecture. This is only a part of the overall process, which includes not only preprocessing, feature selection, and classification operations but also the usual output of confirming high accuracy and robustness. Preprocessing data is the most important aspect of the data pipeline. These stages, normalizing, cleaning, and partitioning the data into training, validation, and test sets, are the ones handled in the backbone of the data pipeline. Data of good quality is the main function of the data, and the data thus purifies the dataset from its noise and avoids sample bias, which are typical requirements for the stages of modeling. Brassy pelican optimization (BPO) algorithm is the one to come in here and explain the feature selection process through the use of one heuristic, which is pelican’s food seeking nature, as an example to go deep into the features with the nature of being informative and distinguishing. More than just reducing the dimensionality of the dataset, this feature also allows the classifier to raise its predictive power by those that are the most suitable for prediction performance.

The Hunting Optimized Recurrent Neural Network–Long Short Term Memory (HO-RNN-LSTM) model is definitely the one which handles the classification task while the Deer Hunting Optimization (DHO) is doing hyperparameter tuning. DHO is like a deer in nature which uses cooperative hunting tactics to explore hyperparameter space sceneries rapidly and choose the best learning rate, the number of hidden units, and many other critical parameters that will result in good convergence, stability, and predictive accuracy. The methodology basically leads to a well-thought pipeline where processing, selecting features as well as classifier optimization are in close cooperation to gain over the challenges of methodological rigor and robustness that are the BOLD framework traits that differ from traditional methods. This work, though, not only points to some merits but also issues like computational efficiency and adaptability to unstructured or highly heterogeneous datasets, which are yet to be solved.

In general, there are different types of preprocessing techniques, like clustering-based, distance-based, z-score normalization, etc. Among other techniques, the min-max normalization technique is increasingly used in the recent healthcare applications for preprocessing. Here, the mean and standard deviation values are computed to generate the normalized values for the original data. In the proposed system, the main purposes of using the min-max normalization technique are listed below:

- To transform the unstructured data into a structured format.
- Formulate the data for scaling.
- It works well for all kinds of data like large, medium and small.
- When compared to other techniques, it is simple to implement.
- Moreover, it maintains the relationships between the original data.

### 3.2. Brassy Pelican Optimization (BPO) for Feature Selection

After preprocessing, the BPO algorithm is used to choose the best attributes from the preprocessed dataset according to the global optimal solution. It is one of the new meta-heuristic models, mimicked by the behavior of the pelican. Its main advantages over other techniques include quick convergence, simple deployment, and little parameter adjustments. They primarily eat fish and have exceptional flying vision and analytical abilities. Once the pelicans have located their prey, they rush at it from an altitude of 10 to 20 m before diving headfirst into the ocean to begin their hunt. The pelican is a huge bird with a long beak and a wide pouch in its throat for catching and swallowing prey. This bird thrives in social situations and flocks of up to a thousand pelicans. Pelicans have the following physical characteristics: average weight between 3 kg and 15 kg, height between 1.06 and 1.83 m, and wingspan between 0.5 and 3 m. Fish makes up the majority of a pelican’s diet, with frogs, sea turtles, and crabs appearing less frequently. If it is really hungry, it may even consume seafood. Pelicans frequently cooperate during hunting. When the pelicans locate their prey, they fly to it at a height of between 10 and 20 m. Naturally, certain species also descend to catch their prey at lower elevations. The fish are then forced into shallow water by the spread of their wings, making it easier for them to catch their prey. The pelican lifts its head forward before swallowing the fish to remove extra water when it catches a fish and a lot of water enters its beak. Pelicans have become proficient hunters as a result of their clever hunting behavior and tactics. The modeling of the aforementioned strategy served as the primary source of inspiration for the conceptualization of the proposed BPA.

Members of the population are initially started at random in accordance with the problem’s lower bound and upper bound. The population matrix is a matrix used to identify the pelican population members in the proposed BPO. The columns that make up of this matrix reflect the suggested values for the problem variables, and each row represents a potential solution. On the basis of each of the potential solutions, the particular problem’s objective value can be assessed. The objective function vector is used to determine the values obtained for the function with an objective. The proposed BPO updates potential solutions by simulating the tactics and behavior of pelicans during attacks and hunts. There are two parts to this hunting technique:

- Exploring the direction of the prey.
- Exploitation phase involves winging on the water’s surface.

In the proposed work, the BPO technique is specifically implemented for reducing the dimensionality of features to ensure an effective classification. In the existing healthcare applications, the different types of optimization techniques are implemented for feature selection and dimensionality reduction. For instance, the Gorilla troop, butterfly algorithm, squirrel search algorithm, and other swarm-intelligence techniques are used in the recent state-of-the-art healthcare applications. When compared to these techniques, the specific reasons of using BPO technique in the proposed study are given below:

- Better competitive performance.
- Improved searching efficacy in the solution space.
- Reduced local optimum.
- Time efficiency.
- Increased convergence rate.
- Balanced exploration and exploitation while optimizing the data.

By using the following equation, the population members are randomly selected at first with the upper and lower bound values:

(1) $$g_{i,j}=L_{j}+f\times \left(U_{j}-L_{j}\right)\;i=1,2,3\ldots m\;\mathrm{and}\;j=1,2,3\ldots n$$

The list of symbols and descriptions is given in Table 2.

Based on the generated population set, the matrix construction is performed as represented in the following model:

(2) $$G={\left[\begin{array}{c} G_{1}\\ \vdots \\ \begin{array}{c} G_{i}\\ \vdots \\ G_{m} \end{array} \end{array}\right]}_{m\times n}={\left[\begin{array}{ccc} g_{1,1} & \begin{array}{ccc} \ldots & g_{1,j} & \ldots \end{array} & g_{1,n}\\ \vdots & \vdots & \vdots \\ \begin{array}{c} g_{i,1}\\ \vdots \\ g_{m,1} \end{array} & \begin{array}{ccc} \begin{array}{c} \vdots \\ \ldots \\ \ldots \end{array} & \begin{array}{c} g_{i,j}\\ \vdots \\ g_{m,j} \end{array} & \begin{array}{c} \ldots \\ \ldots \\ \ldots \end{array} \end{array} & \begin{array}{c} g_{i,n}\\ \vdots \\ g_{m,n} \end{array} \end{array}\right]}_{m\times n}$$

According to the generated matrix, the objective function is formulated as represented below:

(3) $$Z={\left[\begin{array}{c} Z_{1}\\ \begin{array}{c} \vdots \\ Z_{i}\\ \vdots \end{array}\\ Z_{m} \end{array}\right]}_{m\times 1}={\left[\begin{array}{c} Z\left(G_{1}\right)\\ \begin{array}{c} \vdots \\ Z\left(G_{i}\right)\\ \vdots \end{array}\\ Z\left(G_{m}\right) \end{array}\right]}_{m\times 1}$$

Then, the new status of pelican $g_{i,j}^{Y_{1}}$ is updated based on the following model:

(4) $$g_{i,j}^{Y_{1}}=\left\{\begin{array}{cc} g_{i,j}+f\times \left(l_{j}-s\times p_{i,j}\right), & Z_{l}<Z_{i}\\ g_{i,j}+f\times \left(p_{i,j}-l_{j}\right), & else \end{array}\right.$$

Based on the newly updated status of the pelican, the suboptimal position is computed as shown below:

(5) $$G_{i}=\left\{\begin{array}{c} G_{i}^{Y_{1}},\;Z_{i}^{Y_{1}}<Z_{i}\\ P_{i},\;else \end{array}\right.$$

Then, the pelican’s behavior is updated at the time of hunting as represented in the following model:

(6) $$g_{i,j}^{Y_{2}}=g_{i,j}+C\times (1-\frac{itr}{Max\_itr})\times (2\times f-1)\times g_{i,j}$$

According to the newly updated status of the pelican, its best optimal position is estimated, as represented in the following model:

(7) $$G_{i}=\left\{\begin{array}{c} G_{i}^{Y_{2}},\;Z_{i}^{Y_{2}}<Z_{i}\\ G_{i}\;else \end{array}\right.$$

**Figure 3:**
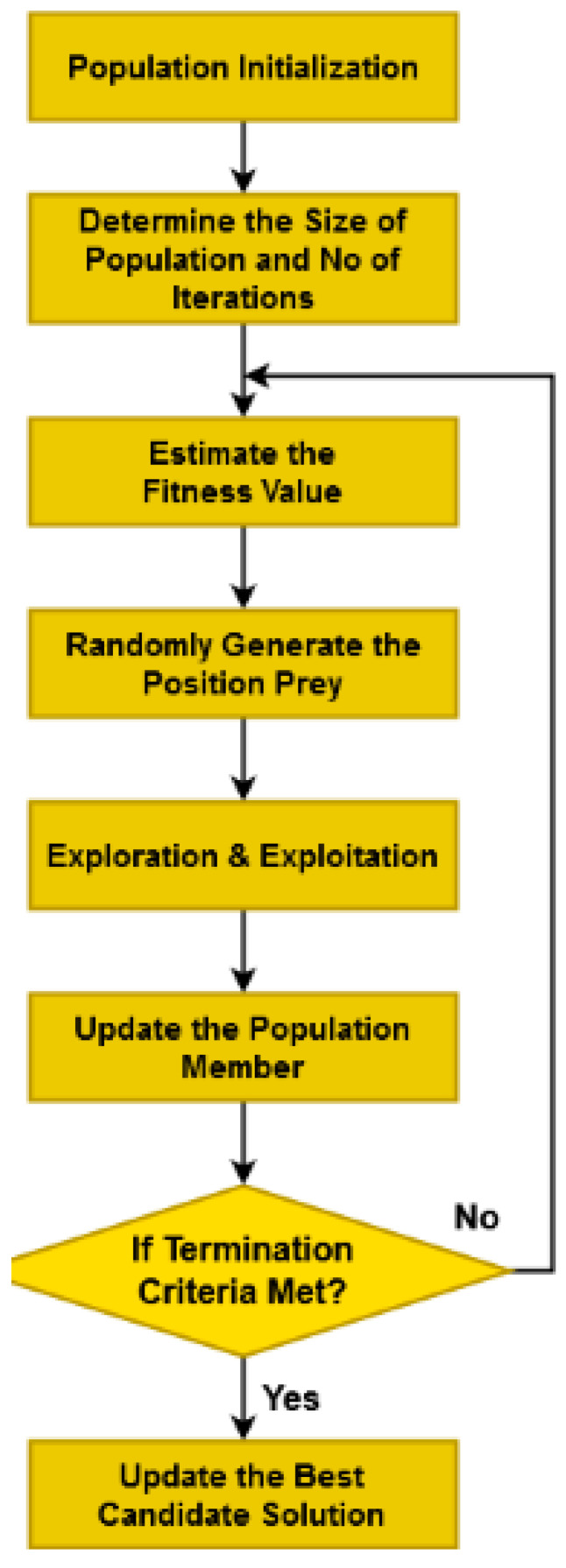
Flow of the BPO algorithm.

The obtained optimal solution can be used to choose the most suitable attributes for minimizing the dimensionality of features. The flow of BPO technique used for feature selection is illustrated in Figure 3 and Algorithm 1 presents the procedure of BPO model.

**Algorithm 1 Brassy Pelican Optimization Algorithm (BPO)**
*Input: Preprocessed dataset;* *Output:  Best optimal solution;* *Step 1: Initialize the optimization problem;* *Step 2: Initialize the total number of populations and maximum number of iterations;*
*Step 3: Randomly generate the population member* 
$g_{i,j}$
 *with upper and lower bounds by using Equation (1);* *Step 4: Determine the position of pelicans with the objective function as shown in Equations (2) and (3);* *Step 5: For* 
itr=1:max_Itr
    *Randomly generate the position of prey*    *For* 
i=1 to m
*      Perform exploration operation;*      *For* 
j=1 to n
 *Update the status of pelican* 
$g_{i,j}^{Y_{1}}$
 *using Equation (4);*        *End for;* *Update the population member* 
$\;G_{i}$
 *as represented in Equation (5);* *Perform exploitation operation;* *For* 
j=1 to n
*Estimate the new status of pelican* 
$g_{i,j}^{Y_{2}}$
 *as represented in Equation (6);* *End for;* *Update the population member* 
$G_{i}$
 *by using Equation (7);*      *End for;*      *Determine the best candidate solution;*    *End;* *Step 6: Result the best candidate solution;*

### 3.3. Hunting Optimized Recurrent Neural Network—Long Short Term Memory (HO-RNN-LSTM)

The HO-RNN-LSTM technique is utilized to accurately categorize the diabetic and non-diabetic patients after selecting the optimum ideal parameters. For diabetic prediction, numerous machine learning and deep learning techniques are applied in current studies. The survey found that the bulk of studies for disease prediction have employed machine learning and an ensemble of machine learning methodologies. However, they are constrained by the main issues of sluggish processing, poor precision, and ineffectiveness. Some deep learning techniques have been used in recent studies to try to solve these issues, but they have their own challenges, such as complicated systems and lengthy training and testing processes for prediction. As a result, the proposed research uses a novel HO-RNN-LSTM model, where the DHO approach is used to tune the hyper parameters of the RNN-LSTM classifier. It aids the classifier in producing accurate illness prediction decisions. Information transmission is controlled by LSTM, which is often described by a sigmoid function, where the cell state resembles a conveyor system in certain ways. The LSTM can modify the cell state by either eliminating or adding information, which is precisely controlled via gates.

In the proposed technique, the HO-RNN-LSTM-based deep learning technique is implemented for predicting diabetes with high accuracy. The proposed HO-RNN-LSTM performs well in comparison to other machine learning and deep learning techniques since it is built on one of the most cutting-edge and effective RNN-LSTM algorithms. Additionally, it can handle large-scale datasets more effectively and requires less time for testing and training. Because the RNN-LSTM incorporates the HO approach for hyperparameter tweaking, the classifier’s efficiency is maximized with excellent prediction accuracy. An input gate and an output gate have the same weight matrices and activation functions. The weighted input and output of the input gate can stop the cell from receiving extraneous data. The output gate modifies the cell’s output according to its weighted input in a similar way. An input layer, an output layer, and a self-connected hidden layer collectively make up an LSTM cell. The hidden unit may contain the ‘conventional’ units that can be supplied into subsequent LSTM cells. However, due to its linear structure, a typical LSTM cell also complies with some limitations. It was concluded that if it kept growing, it might reach function saturation and become an ordinary unit. As a result, another forget gate layer was included. A new gate makes it possible to erase and forget unnecessary information. An LSTM cell with a forget gate behaves similarly to a standard LSTM, with one exception.

The function of the gate layer is to select the top layer of input data that may be deleted. It also controls the hidden layer nodes that are kept in the most recent historical data. The forget gate operates on the preceding state of the cell to figure out the data that has to be maintained and what information ought to be wiped out. It estimates a value ranging from zero to one based on the hidden layer’s previous state and the input of the present-time node. The processing function of the forget gate allows for the selective processing of the output of the hidden layer cell (historical data). After obtaining the input features, the hidden state function is computed as shown in the following model:

(8) $$F_{t}=\varphi (\omega_{F}\times \left[H_{t-1},X_{t}\right]+B_{F})$$

where $F_{t}$ indicates the forget gate, φ is the sigmoid function, $\omega_{F}$ represents the weight value of forget gate, $H_{t-1}$ denotes the input, $X_{t}$ is the expected output, and $B_{F}$ is the bias value. The input cell state of the hidden gate layer is controlled by the output gate layer. It can input the data using a variety of processes to decide what needs to be preserved to change the state of the cell at the moment. A sigmoid function is used to initially construct the input gate layer and determine which information has to be adjusted. The updated value is then calculated by combining both together by initially creating a tanh layer and inserting the prospective state of the neuron phase. The input gate and candidate state are determined by using the following equations:

(9) $$I_{t}=\varphi (\omega_{I}\times \left[H_{t-1},X_{t}\right]+B_{I})$$

(10) $${\tilde{C}}_{t}=tanh(\omega_{C}\times \left[H_{t-1},X_{t}\right]+B_{C})$$

where $I_{t}$ is the input gate, ${\tilde{C}}_{t}$ indicates the condition state, $\omega_{I}$ and $\omega_{C}$ are the weight values of the input gate and condition state, $B_{I}$ and $B_{C}$ are the bias values of the input gate and condition state, and tanh(.) is the tanh layer. During the neuron state update, the old cell state is updated with the new cell state, where the previous state is multiplied by the forget gate. The neuron state update is performed by using the following model:

(11) $$C_{t}=F_{t}{\times C}_{t-1}+I_{t}\times {\tilde{C}}_{t}$$

Finally, the output of the current hidden layer is controlled by the output layer, which predicts the information to be displayed as the output. The output produced by the output layer is determined by the following models:

(12) $$O_{t}=\varphi (\omega_{O}\left[H_{t-1},X_{t}\right]+B_{O})$$

(13) $$H_{t}=O_{t}\times tanh(C_{t})$$

Here, the activation function φ is optimally computed by using the DHO technique, which helps to increase the accuracy of prediction with low system complexity.

### 3.4. Deer Hunting Optimization (DHO) for Parameter Tuning

In this work, the DHO algorithm is mainly applied to speed up the process of the classifier by supporting it to make an effective decisions while predicting diabetes. In deep learning classification, hyper parameter tuning is typically crucial since it raises the classifier’s overall prediction accuracy. Even though the primary goal of the suggested algorithm is to identify the best location for a hunter to stalk a deer, deer behavior must first be understood. They possess certain traits that make it challenging for predators to hunt them. One of these qualities is the visual ability, which is five times greater than that of a human. However, they have difficulty seeing the hues red and green. A deer, often known as a buck, can detect even the slightest movement, and according to biologists, a white-tailed deer’s field of vision is between 250 and 270 degrees. This makes it easier for a buck to track the hunter’s movements beneath the horizon. After the best positions are established, everyone in the population works to get the best position, which starts the process of updating the position. The idea is expanded by taking the position angle into account in the update rule to broaden the search space. Calculating the angle is crucial for positioning the hunter so that the prey is unaware of the attack and the hunting procedure is successful. A parameter is computed that aids in updating the location angle based on the difference between the wind angle and the deer’s visual angle. By changing the vector during the exploration phase, a similar concept in encircling behavior can be implemented. The value of the considered vector is less than 1, as we initially presumptively conduct a random search. As a result, rather than using the initial best solution found, the position update depends on the next-best position.

Using the DHO approach, the activation function is here best calculated. The DHO technique has several advantages over other optimization algorithms, including a higher rate of convergence, faster searching, and a reduced number of iterations needed to find the best solution. Therefore, the proposed work uses the DHO technique for tuning the parameter of RNN-LSTM. It is inspired by the hunting behavior of deer, where the cooperation between the hunters is considered as an important criterion, since it supports an effective hunting operation. Typically, the deer are able to readily avoid being hunted by predators because of their unique talents. A vector of the hunters, a random population, serves as the algorithm’s initial input. The following model is used to determine this process:

(14) $$K=\left\{K_{1},K_{2}\ldots K_{n}\right\}\;\;1<j\leq n$$

where n indicates the number of hunters, and K is the set of population. In this technique, some of the important parameters such as position and wind angle are estimated in the searching space. It is defined in the following equation:

(15) $$\theta_{j}=2\pi \delta$$

where $\theta_{j}$ represents the wind angle, and δ is the random number ranging from 0 to 1. Then, the position propagation is performed for the successor and leader according to the weight values. During the leader position propagation, the best position is initialized, where each population tries to reach the best optimal location. According to the encircling behavior, the position update is performed by using the following equation:

(16) $$K_{j+1}=K_{L}-M\times \beta \times |l\times K_{L}-K_{j}|$$

where $K_{j}$ indicates the position of the deer at the current iteration, $K_{j+1}$ represents the position of the deer at the subsequent iteration, and β is the random number between the range of 0 to 2. Then, the coefficient vectors are computed by using the following models:

(17) $$\begin{array}{l} C1=\frac{1}{4}log\left(j+1/j_{m\_itr}\right)h\\ C2=2\times C \end{array}$$

where C1 and C2 are the coefficient vectors, m_itr represents the maximum number of iterations, and h indicates the random value ranging from −1 to 1. Then, the propagation through position angle is estimated in order to enable an effective hunting process. It is performed by using the following equation:

(18) $$K_{j+1}=K_{L}-\tau \times |cos(\vartheta )|\times K_{L}-K_{j}|$$

where τ indicates the random number. Moreover, the successor position is estimated during the stage of exploration, and the global search is performed by using the following model:

(19) $$K_{j+1}=K_{s}-C2\times \tau \times |C1\times K_{s}-K_{j}|$$

where $K_{j+1}$ is the updated best position, and $K_{s}$ indicates the successor position. By using the position update, the best optimal solution is identified, which is used to tune the activation function of the classifier. Generally, the deep learning techniques are more complex to implement, since it requires difficult mathematical calculations for accomplishing classification. Among other deep learning models, the RNN-LSTM is significantly better, and it performs classification with simple calculations. Yet, the time taken by the RNN-LSTM for training and testing operations is slightly higher. In the proposed work, this problem is resolved by applying the HO technique to tune the hyper parameter to make accurate decisions with less computational time. The DHO algorithm is one of the most significant and recent optimization algorithms, and is well-suited for solving some problems with its simplified solutions. Among other algorithms, the specific reasons and benefits of applying the DHO technique for parameter tuning are given below:

- It simplifies the process of classification by providing the best optimum value to tune the hyper parameter.
- Increased searching efficiency.
- Suitable for handling complex data.

The process by which a computing system picks up characteristics from input data is called learning from experience. Such methods have shown to be successful in identifying diabetes.

Given that predictive techniques are data-driven, this is obviously practicable. With so much data being entered into the database, predictive modelling can significantly reduce the need for human effort. These data are used for training models, which then use the input data to determine the best possible output. Any set of practical and medically necessary parameters can be used to train the models. While some might look at features, others might search for patient blood report data. The parameters change as a result of the disease’s numerous symptoms. Using a variety of prescribed techniques, researchers have examined several algorithms and adjusted a great deal of hyperparameters to produce findings that are most appropriate for practical uses.

## 4. Results and Discussion

This section uses a number of performance indicators to carry out a clear performance assessment and comparison. In order to put the suggested Deep Harmony Learning (DHL) technique into practice, a system must be configured with the right hardware and software. A multi-core CPU, lots of RAM (16 GB, for example), quick storage (SSD, for example), and, if desired, an NVIDIA GPU with CUDA capability for faster calculations are the minimum hardware requirements. Python 3.7 or higher must be installed on Ubuntu 18.04 LTS or later to function as the recommended operating system. NumPy, Pandas, TensorFlow, PyTorch, and Scikit-learn are among the essential Python 3.11 libraries needed for deep learning model building and assessment, as well as for preprocessing data. Acquiring datasets include locating pertinent datasets, such as the PIMA Indian Diabetes Dataset (PIDD), and establishing a directory structure to arrange models, code, data, and outcomes. Preprocessing, feature selection, model training, hyperparameter tuning, and other tasks are developed as scripts. In this study, several public datasets such as Indonesia diabetic database, PIDD, and kidney disease dataset have been used for testing and analysis. A well-known and frequently used dataset for the diagnosis of diabetes is the Pima Indian Diabetes Database (PIDD). There are 9 columns and 768 rows in this particular set of data with the characteristics of glucose, pregnancies, skin thickness, blood pressure, BMI, insulin, and age [41]. The performance assessment parameters including sensitivity, specificity, precision, and accuracy are used to determine the method’s success, which are computed by using the following equations:

(20) $$Accuracy=\frac{T_{Pos}+T_{Neg}}{T_{Pos}+T_{Neg}+F_{pos}+F_{Neg}}\times 100\%$$

(21) $$Precision=\frac{T_{Pos}}{T_{Pos}+F_{Pos}}\times 100\%$$

(22) $$F1-score=\frac{2\times Pre\times Sen}{Pre+Sen}\times 100\%$$

(23) $$Recall=\frac{T_{Pos}}{T_{Pos}+F_{Neg}}\times 100\%$$

(24) $$Sensitivity=\frac{T_{Pos}}{T_{Pos}+F_{Neg}}\times 100\%$$

(25) $$Specificity=\frac{T_{Neg}}{T_{Neg}+F_{Pos}}\times 100\%$$

where $T_{Pos}$ —true positives, $T_{Neg}$ —true negatives, $F_{Pos}$ —false positives, and $F_{Neg}$ —false negatives. The effectiveness of the prediction algorithms is visualized using a confusion matrix, which cross calculates the actual and forecasted classes with related values.

The performance metrics of the BOLD framework were tested in real time and cross-verified through multifarious data processing and validation methods to ensure that the metrics are dependable and can be extended to various other situations. These actions were implemented to rule out the confounding of overfitting, data leakage, or biased evaluation. Primarily, the datasets for the research, i.e., Pima Indian Diabetes Dataset (PIDD), Indonesia Diabetic Database, and Chronic Kidney Disease Dataset, were individually preprocessed to eliminate cross-contamination among the datasets. Each dataset was partitioned by an 80–20 ratio, where 80% of the samples were utilized for training, and 20% were for testing. Moreover, stratified random sampling was applied for partitioning, so the class distribution was the same as in the original data, and there was no bias towards the majority class.

To increase the power of the model and to avoid overfitting the training subset, a 10-fold cross-validation method was employed at both the feature selection and hyperparameter optimization levels. The procedure entailed the division of the dataset into ten subsets of equal size. In each of the ten iterations, the training was done on nine folds, and the validation was done on the remaining one fold. They conducted the process ten times, and the averaged results were used as the final model performance. The cross-validation procedure they referred to was the one in which each and every data point in the dataset was not only used for training but also for validation in different iterations; hence, the model could not associate certain patterns or noise with one data split.

The optimization of the BPO algorithm that was used for the feature selection was done only on the training folds. After that, the features picked were the ones used in the validation and test folds that were new to the model. In such a fashion, the validation and test sets were truly independent ones and, hence, they had not been exposed to the optimization or learning processes beforehand. Furthermore, the nested cross-validation technique was used at the Deer Hunting Optimization (DHO)-based hyperparameter tuning of the RNN–LSTM classifier. The inner loop of this nested configuration was focused on the parameter DHO-based search, whereas the outer loop was verifying the tuned model on the different holdout fold. This hierarchical validation structure allowed for a fair, unbiased, model generalization estimate and, hence, the likelihood of model overfitting due to hyperparameter tuning on the same data used for performance evaluation was very much limited. Besides this, overfitting during the LSTM training was also alleviated by early stopping and dropout regularization with the dropout rates of between 0.2 and 0.5 discouraging the network from co-adapting to certain neuron patterns.

Furthermore, to ensure the model’s soundness across different datasets, the model was separately trained and tested on each dataset; thus, the datasets were not pooled together into one big corpus that could have caused unintentional bias or the overlap of the feature-space. Hence, the high performance reported, e.g., an accuracy of 0.996 and AUC of 0.99, was not the single evaluation run but rather the cross-validation average of multiple runs which were stable across datasets. Moreover, the performance was compared to that of the traditional models like SVM, Random Forest, Gradient Boosting, and baseline LSTM networks which were using the same cross-validation splits. Thus, the performance enhancement was at the level of statistical significance (*p* < 0.05). All these methodological steps combined together serve as a guarantee of the proposed BOLD framework results being not only reproducible and reliable but also that they are hardly susceptible to overfitting or data leakage—the major reasons why the claim of its diagnostic efficiency for diabetes prediction in real-world clinical data scenarios is becoming stronger.

The BOLD brass optimized learning-based diabetes prediction framework is a next-generation diabetes prediction method. One of its goals is to be the only method for diabetes prediction. With such a goal in mind, it is quite normal that their system is conducted in a very transparent way and at the highest level of reproducibility. Thus, the whole machine has been unveiled as a multi-staged pipeline. Moreover, the authors kept their word from the last study and similarly, they made a decision to utilize the vast community and libraries available for Python by the implementation of the entire pipeline in Python 3.9. The deep learning parts were created with the help of TensorFlow 2.x and Keras. The remainder of the pipeline, however, was done with NumPy, Pandas, and Scikit-learn, which were used for preprocessing, normalization, and evaluation, respectively.

This work was equipped with the activities of discarding missing entries, outliers, redundant attributes, etc. They went on to standardize the data through a min-max scaling technique which brings all the features to the [0, 1] interval. Furthermore, the data were divided into a training subset (80%) and a test subset (20%). The split was done using a stratified sampling method which enabled the balancing of classes. The feature optimization script for Brassy Pelican Optimization (BPO) was created in such a manner that it can be understood that the running of the module means simply running the BPO algorithm with a pelican population of 40 and max iteration 150 already set. One pelican stands for one possible feature set out of which each pelican is a binary vector, with ‘1’ meaning that the feature is included and ‘0’ that the feature is excluded. The fitness function that BPO utilizes is the one which returns the highest value of classification accuracy. The testing will be extremely fast; at the same time the link between the feature subset and the classifier’s performance will be kept due to this idea.

Then, the Hunting-Optimized RNN-LSTM classifier was trained with the chosen features. The DHO operation was started with a herd of deer moving randomly in the search space, thus each one representing a different configuration of learning rate (range 10^−5^–10^−2^), batch size (16–128), number of LSTM units (32–256), and dropout (0.1–0.5). The DHO iterative process was similar to the natural one; animals get separated in the group movement stage (exploration); in the coordinated hunting stage (exploitation), they concentrate on the target. This was also instrumental in the exact identification of the hyperparameter configuration leading to the lowest RMSE on the validation data. Model convergence was tracked by early stopping with a patience of 10 epochs and at the same time, overfitting was prevented with the model being finally trained for a maximum of 200 epochs, with the Adam optimizer.

Initially, for a proper assessment of the BOLD framework’s effectiveness, the evaluation datasets were divided into three separate groups: training, validation, and testing sets. The data was partitioned in such a way that 70% was assigned to training, 15% to validation, and 15% to testing. This interaction enables the model to not only take in the features of the training data but also employ the validation set for updating the hyperparameters of the Deer Hunting Optimization (DHO) technique. On top of that, the k-fold cross-validation (k = 10) was utilized at the training stage in order to exclude any overfitting situations and maintain the generalization aspect. As a result, the model was tested on different data portions, thus making the evaluation more dependable. Besides data partitioning, which was executed appropriately, the new approach was pitted against several benchmark algorithms including Support Vector Machine (SVM), Random Forest (RF), K-Nearest Neighbors (KNN), Naïve Bayes (NB), Logistic Regression (LR) as well as conventional deep learning architectures like CNN and standard LSTM models. The evaluation of the models was carried out by using the metrics of accuracy, precision, recall, F1-score, and AUC, which indicate the deeply examined model effectiveness. The results demonstrated that the BOLD framework was not only successful in surpassing the baseline methods for all the benchmark datasets where the experiments were carried out but even maintained its performance levels over time; thus, both the stability and reliability in forecasting were confirmed. This comprehensive experimental and comparative survey is among the main factors that signal the novelty and the extent of the proposed method.

Despite the fact that the suggested BOLD framework mostly reflects the excellent predictive abilities of the various datasets, it is still important to give a more balanced and critical view of the results by discussing its several limitations. The datasets, i.e., PIDD, the Indonesian diabetic dataset, and the kidney disease dataset, which have been used in the experiments, although quite standard among the community of researchers, may not be adequate to represent clinical variability, genetic diversity, and lifestyle factors that exist in different populations distributed globally, which will then affect the transferability of the model. Additionally, in this case, the work is done only on the structured datasets, while the healthcare data of the world is known to be unstructured, such as electronic health records, clinical notes, or sensor data, which are new places for the model. Besides that, the issue of bias due to the distribution of class imbalance may still exist, and even with the use of preprocessing and validation techniques, the bias may continue to have an impact on the results. Though indeed multiple performance metrics and various statistical analyses have been considered, the properties of the framework through time, its recognizability by clinicians, as well as being of practical use in a clinical setting and the successful healthcare system integration, can just be verified by the follow-up validation. Such limitations signal the areas of further work required for the system improvement, more comprehensive datasets, and actual testing for easier access and system robustness.

In this study, some of the baseline models [42] including Logistic Regression (LR), Naïve Bayes (NB), Decision Tree (DT), Nearest Neighbor (NN), Random Forest (RF), Cat Boost (CB), Ada Boost (AB), Gradient Boost (GB), Bagging classifier, and voting model are considered for comparative analysis. These are all the most commonly used classification approaches used in conventional works for the prediction and diagnosis of diabetes. Moreover, the detection results and efficacy of these techniques are assessed and compared according to the performance measures as described above. In the proposed study, PIDD—medical data—which includes patient information with various features including age, gender, blood glucose level, BMI, and others—is gathered from public sources.

The datasets for both training and testing of the BOLD framework, namely the PIDD, Indonesian diabetic dataset, and kidney disease dataset, are very stable and recognized by the majority of researchers in the field of chronic disease prediction as an appropriate basis for the development and evaluation of the model. These datasets are a combination of patient demographics, clinical attributes, and disease-related features from diverse sources, which the model can extract to learn the occurrence patterns of diabetes and its associated complications. Nevertheless, the results that are given here show that the model performs well on these datasets, but the extent to which the model can be generalized to entirely new populations or datasets with different feature distributions may still be a problem that necessitates further validation and probably adaptation. At the same time, the deployment of different datasets and a wide range of evaluation metrics give us an indication that the BOLD framework is likely to be strong enough for application in different healthcare scenarios.

### Dataset Description

The PIDD dataset might be used to validate and compare the findings of the BOLD framework, and performance is evaluated in terms of accuracy, ROC, precision, recall, and f1-score. The PIDD dataset might be used for technique validation and evaluation in the majority of earlier investigations in the field of diabetes or chronic illness diagnosis. Additionally, the accuracy, precision, and recall characteristics of the classifier are used to validate its detection ability. As a result, the proposed study makes use of these metrics to confirm the effectiveness of the suggested method. The PIDD is one of the most emerging and publicly available datasets extensively used in many remote healthcare applications for diabetes diagnosis. In the proposed framework, the original input dataset is directly obtained from public repositories, where the mining operations, such as preprocessing, feature selection, and classification operations are carried out to make accurate predictions about diabetes. The PIMA dataset comprises the following attributes, and its statistical analysis is given in Table 3:

1. Pregnancies: Number of times pregnant.
2. Glucose: Plasma glucose concentration per 2 h in an oral glucose tolerance test.
3. Blood Pressure: Diastolic blood pressure (mm Hg).
4. Skin Thickness: Tricep skin fold thickness (mm).
5. Insulin: 2 h serum insulin (mu U/mL).
6. BMI: Body mass index (weight in kg/(height in m)^2^).
7. Diabetes Pedigree Function: A function that scores likelihood of diabetes based on family history.
8. Age: Age (years).
9. Outcome: Class variable (0 or 1), 1 indicates diabetes, 0 indicates non-diabetes.

Here, mean indicates the central tendency of the data, standard deviation shows the amount of variation or dispersion in the dataset, minimum and maximum the range of values in the dataset.

**Figure 4:**
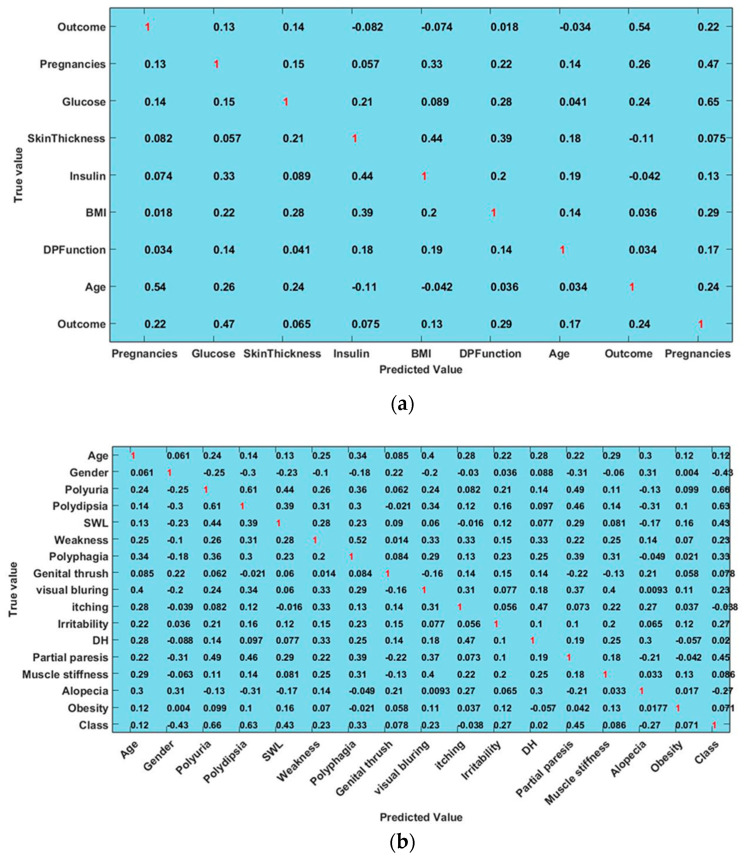
(**a**) Correlation matrix. (**b**) Correlation matrix with different attributes.

The validation of the correlation matrices produced by the BOLD prognostic system using the PIDD dataset is shown in Figure 4a and Figure 4b. The true and prediction values are used to group the classes in this instance. Overall findings show that the BOLD framework precisely separates the classes to produce excellent predicted results. The proposed BOLD architecture significantly increases the classifier’s overall effectiveness by incorporating BPO and DHO approaches.

**Figure 5:**
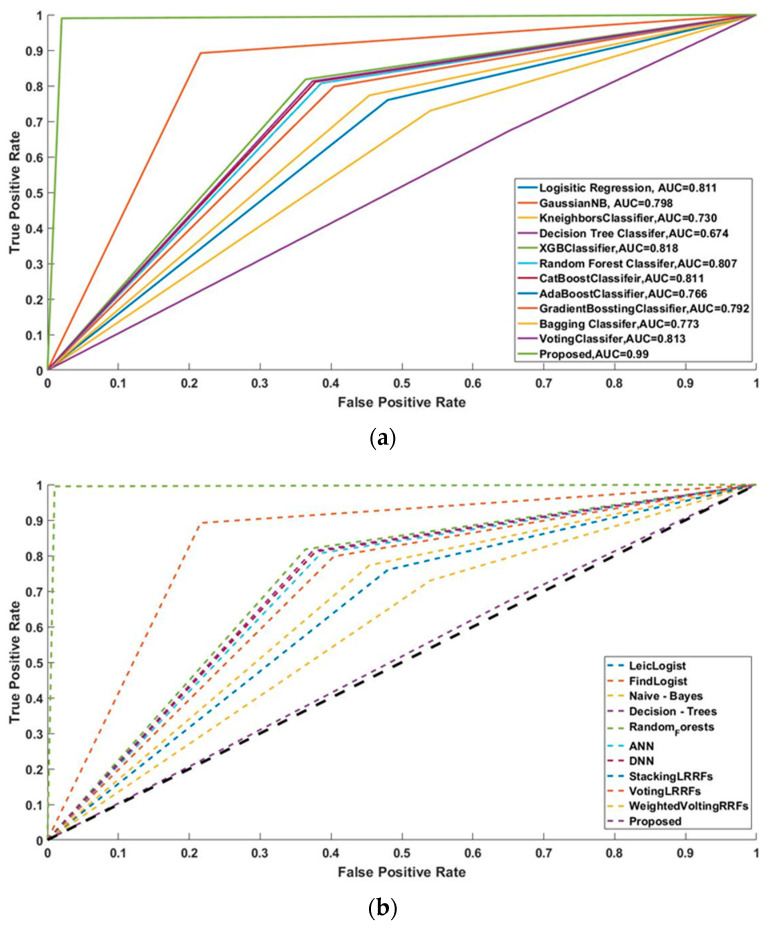
(**a**) ROC comparison with machine learning techniques. (**b**) ROC comparison with deep learning techniques.

Additionally, as shown in Figure 5a,b, the Receiver Operating Characteristics (ROC) are computed and contrasted with machine learning and deep learning techniques. The performance of an efficient classifier is typically assessed using the enhanced ROC analysis. Furthermore, the TPR and FPR values of ROC are used to evaluate the choices made by the weak and strong classifications. As a result, the classifier’s increased ROC shows accurate system performance and results. According to comparison estimates, the ROC of suggested BOLD will be significantly higher when compared to machine learning and deep learning techniques.

**Figure 6:**
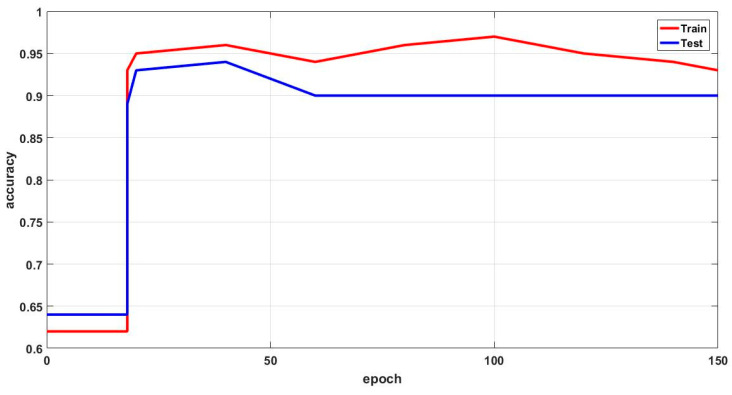
Training and testing performance analysis.

The BOLD framework’s training and testing accuracy performance is validated and compared as shown in Figure 6. According to its improved training and testing performance rate, the classifier’s prediction performance is evaluated. The results show that the inclusion of BPO and DHO approaches significantly increases the training and testing accuracy of the HO-RNN-LSTM technique in the proposed system, since choosing the optimum attributes and fine-tuning hyperparameters is crucial for improving performance in the suggested model.

**Figure 7:**
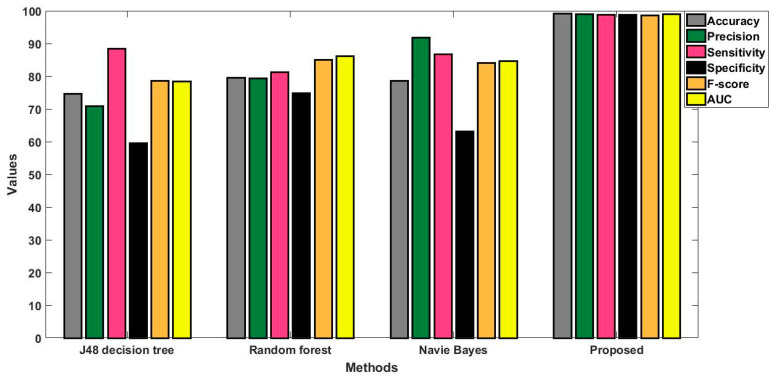
Overall performance analysis with the standard machine learning techniques used for diabetes prognosis.

**Figure 8:**
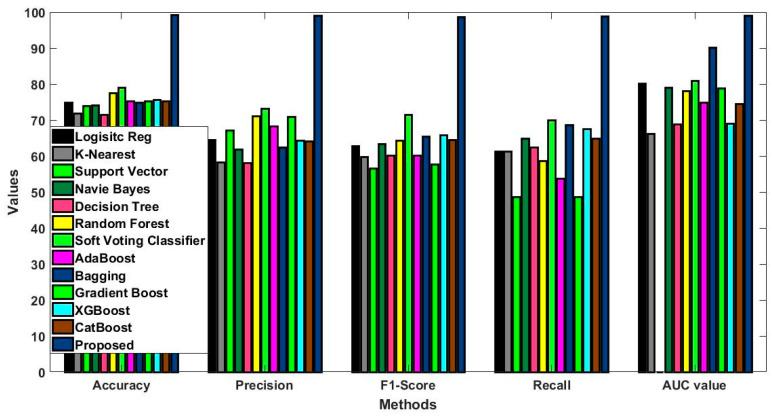
Performance comparative analysis with other machine learning algorithms.

The performances of several machine learning models used for diabetes prognosis as well as the common machine learning approaches that are frequently employed in literature studies [43] are compared in Figure 7 and Figure 8, respectively. This study validates and compares the accuracy, precision, sensitivity, specificity, f1-score, and AUC parameters for this evaluation. The results obtained show that the suggested BOLD outperforms other machine learning models with better outcomes. The BOLD model can produce good results with the right training and testing procedures.

**Figure 9:**
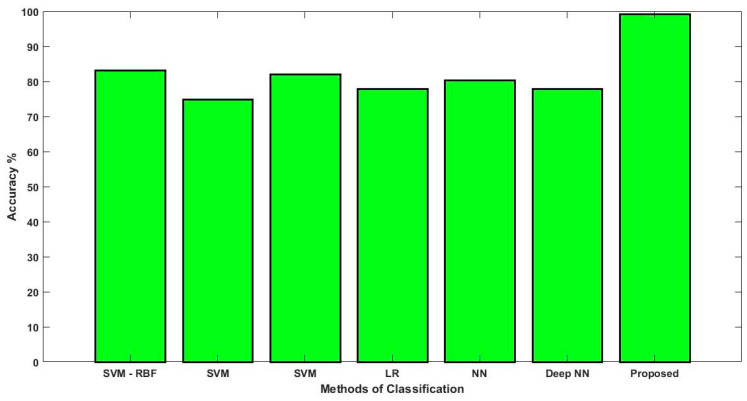
Accuracy of different classification techniques.

**Figure 10:**
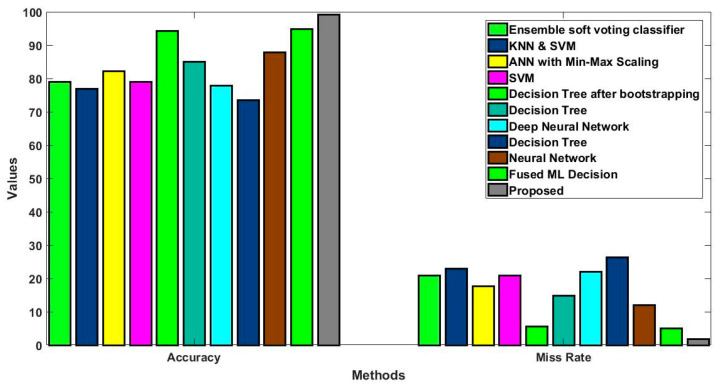
Accuracy and miss rate analysis.

**Figure 11:**
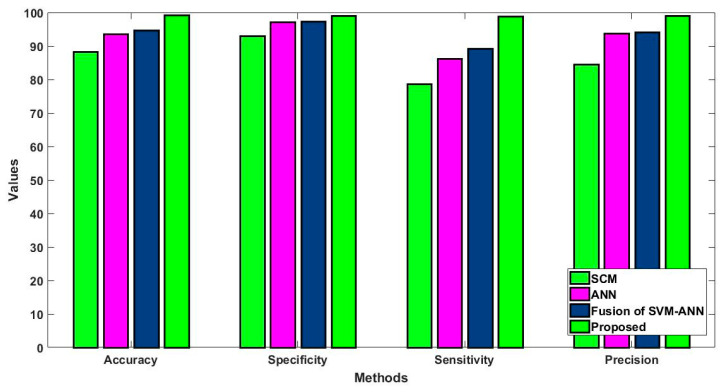
Comparison with fusion models.

**Figure 12:**
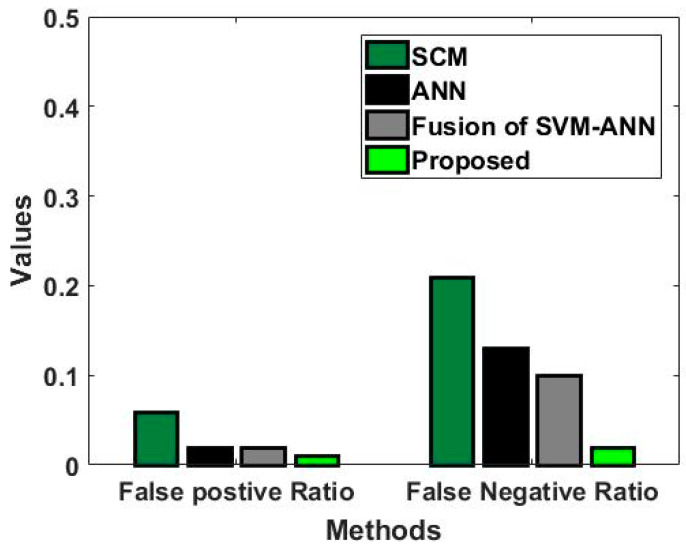
FPR and FNR.

**Figure 13:**
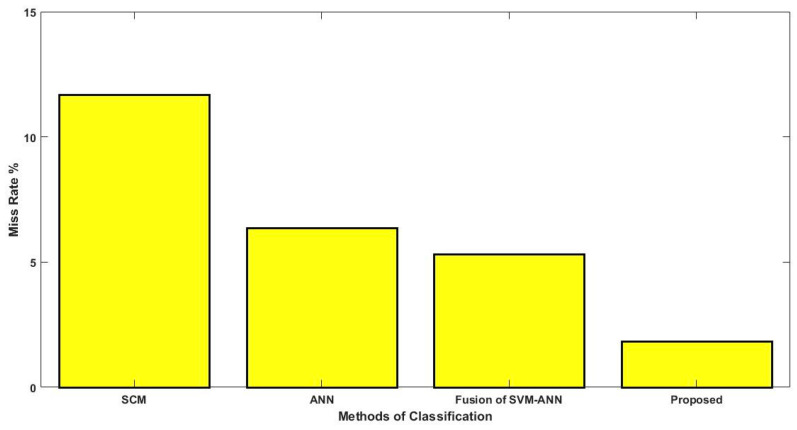
Miss rate.

The classification accuracy and miss rate of different algorithms [44] used for diabetes prognosis are validated and compared in Figure 9 and Figure 10. The suggested model includes a number of mining operations that help to increase detection performance, including cleaning, normalization, splitting, attribute selection, hyperparameter tweaking, and prediction. Additionally, the findings obtained are far superior to those of the earlier models. Moreover, some of the fused models [45] are also compared with the proposed BOLD technique, as shown in Figure 11, Figure 12 and Figure 13.

**Figure 14:**
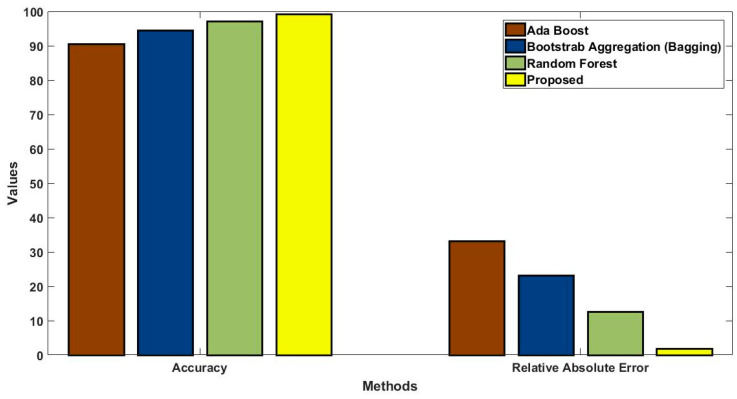
Accuracy and RAE.

**Figure 15:**
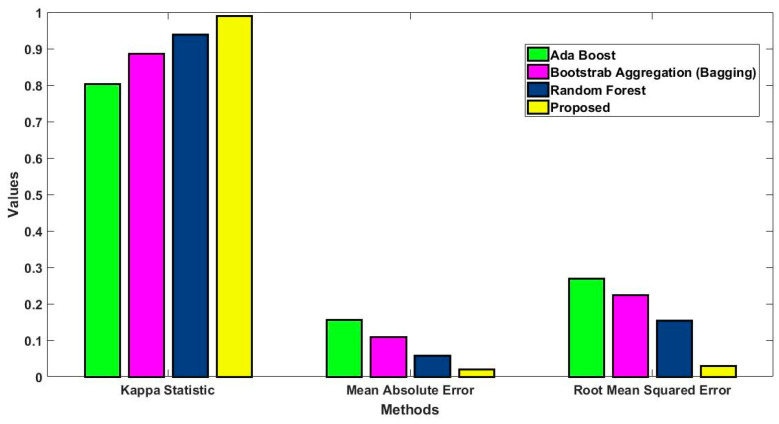
Similarity coefficients.

**Figure 16:**
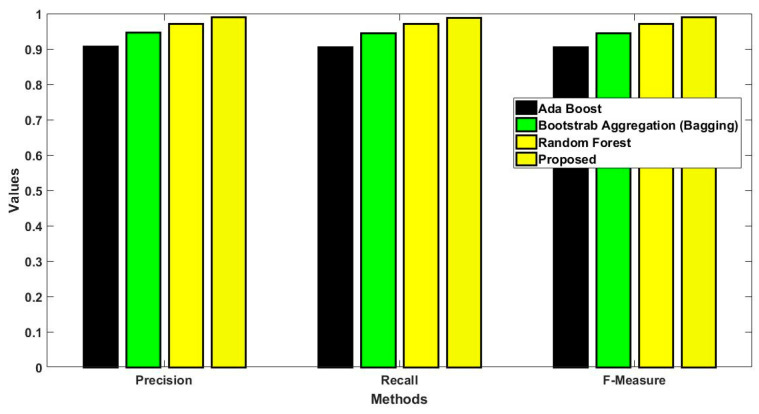
Precision, recall and f-measure rate.

**Figure 17:**
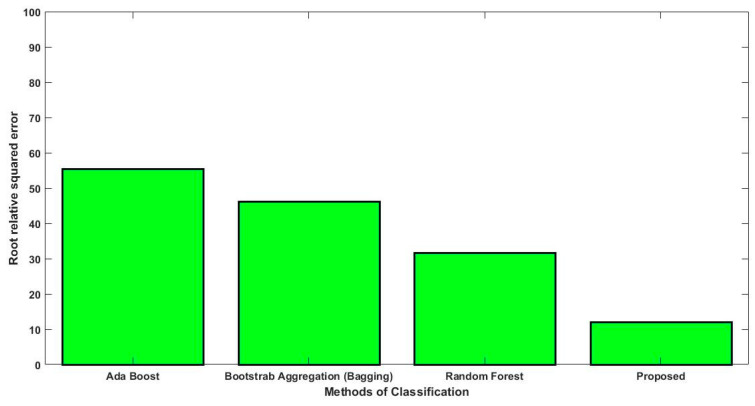
Root Relative Squared Error (RRSE).

**Figure 18:**
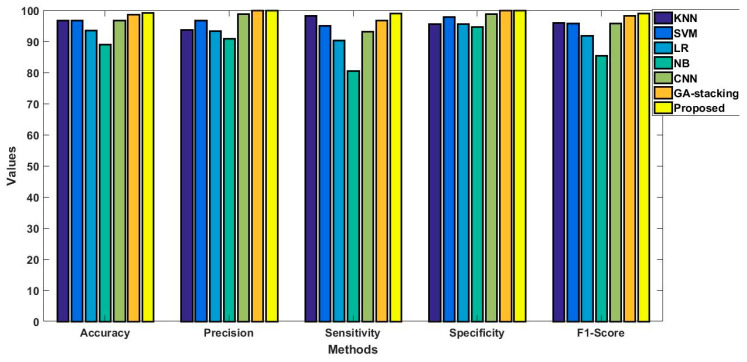
Performance comparison with optimization-based classification models.

Figure 14, Figure 15, Figure 16 and Figure 17 validate and compare the performance outcomes of existing Ada boost, Boostrab aggregation, RF [46] and the proposed BOLD techniques, where the similarity coefficients and error rate are also computed in order to determine the superiority of the classifier. Similarly, some of the optimization-integrated classifiers are contrasted with the BOLD model as shown in Figure 18. According to the findings, it is revealed that the proposed BOLD technique provides accurate prognosis outcomes by perfectly categorizing the diabetic and non-diabetic patients according to their selected data attributes. Effective medical dataset handling plays a vital role in the remote healthcare system, since an inaccurate prediction could make the entire system go down. Therefore, applying proper mining operations can be more helpful for accurate disease prediction. Due to this fact, the proposed BOLD framework includes a group of mining stages for diabetic prognosis.

**Figure 19:**
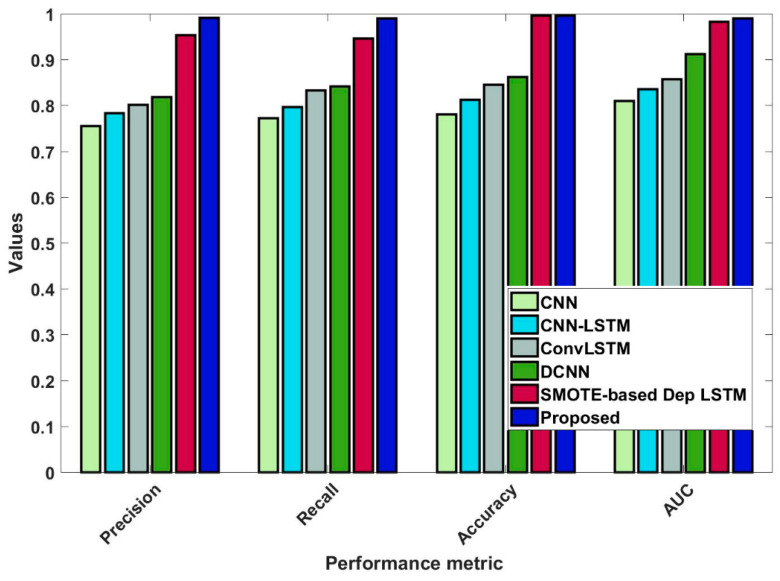
Comparative study with deep learning techniques using Indonesia diabetic database.

**Figure 20:**
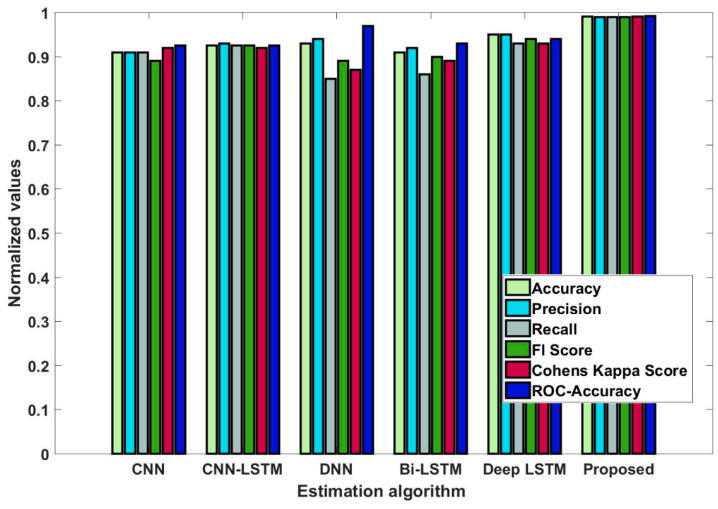
Overall comparative analysis with hybrid deep learning techniques using kidney disease dataset.

**Figure 21:**
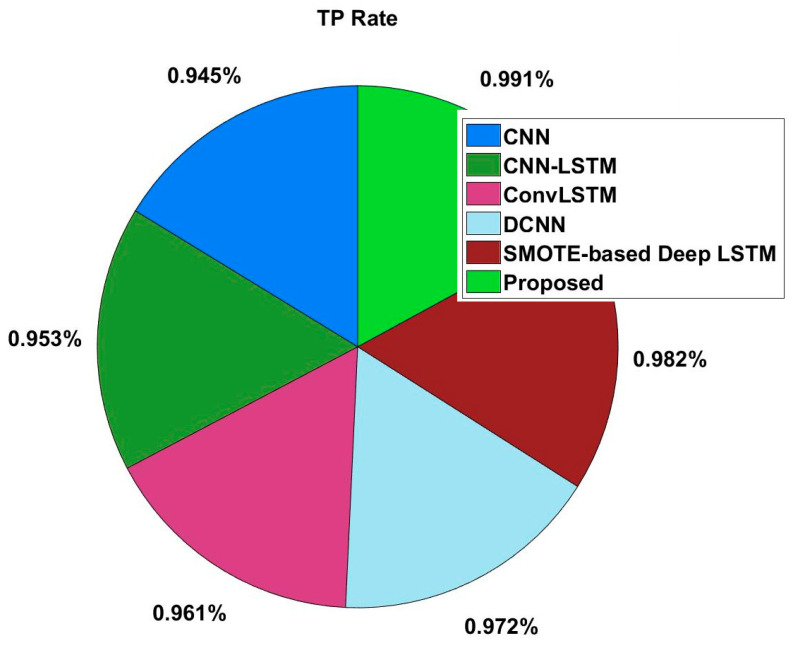
TPR analysis using Indonesia diabetic database.

**Figure 22:**
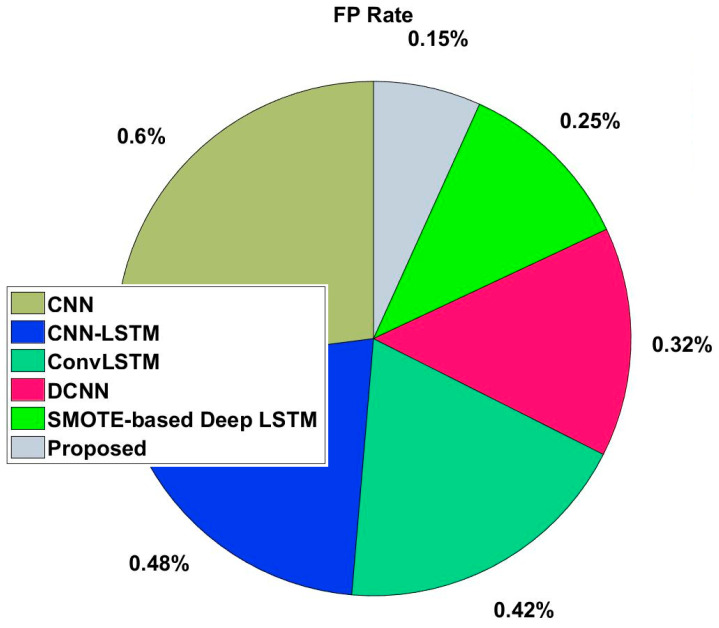
FPR analysis using Indonesia diabetic database.

**Figure 23:**
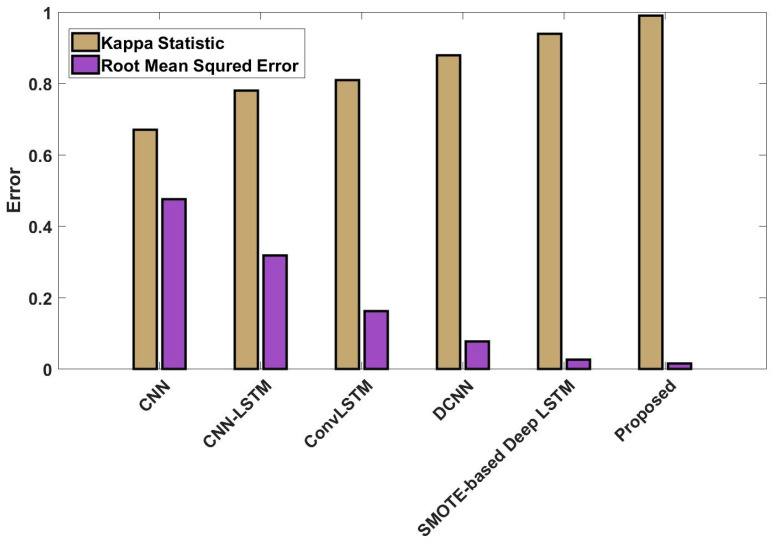
RMSE and Kappa statistical analysis using Indonesia diabetic database.

Using the PIMA dataset, Figure 19 and Table 4 compares the precision, accuracy, recall, and AUC values of the suggested and traditional classification algorithms using Indonesia diabetic database. Likewise, as illustrated in Figure 20, the hybridized deep learning methods are also verified and contrasted with the suggested classifier using kidney disease dataset. This investigation shows that, in comparison to alternative methods, the suggested BOLD model yields better performance results. Furthermore, the suggested BOLD model is contrasted using Indonesia diabetic database with parameters like TPR and FPR, as illustrated in Figure 21 and Figure 22, respectively. Following that, as shown in Figure 23 and Table 5, the RMSE and kappa metrics are validated for the same techniques using Indonesia diabetic database. These results show that the BOLD outperforms all current approaches in terms of precision, recall, accuracy, kappa, f1-score, and decreased RMSE, since the suggested framework’s enhanced results are primarily the result of the application of intelligence algorithms.

**Figure 24:**
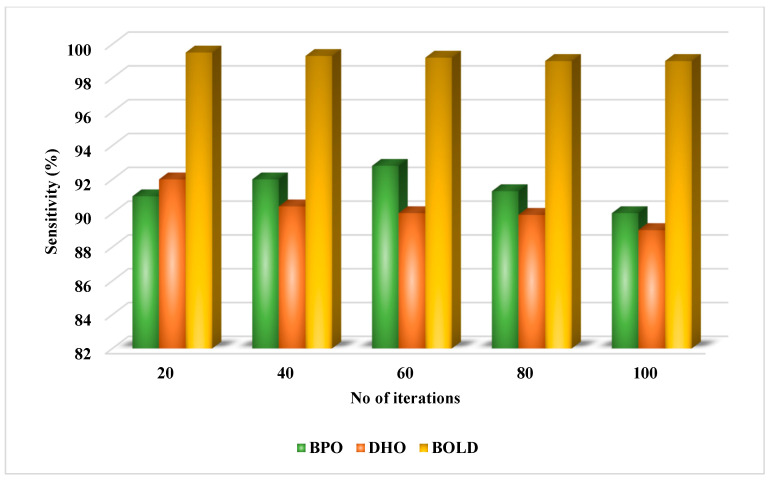
Sensitivity analysis among the optimization techniques.

**Figure 25:**
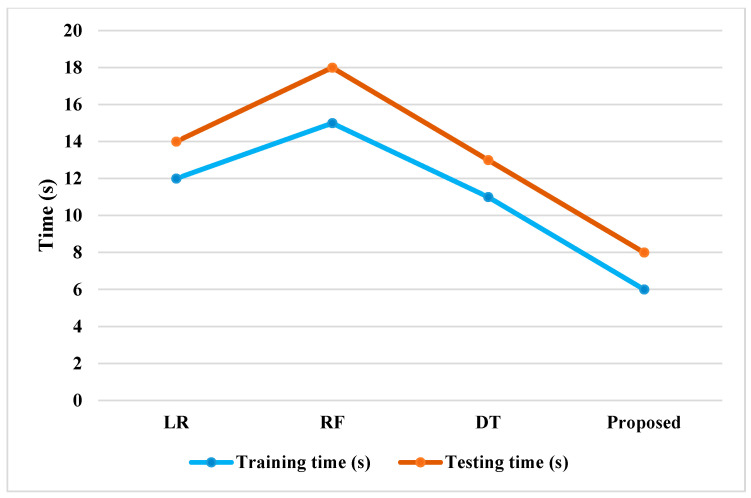
Time complexity using kidney disease dataset.

Figure 24 validates and compares the sensitivity of the BPO, DHO, and BOLD techniques with respect to changing iterations. To determine the efficacy of hybridization, these techniques are compared based on their level of sensitivity. From the results, it is concluded that the proposed BOLD outperforms other techniques with high performance results. In order to calculate the overall time complexity of illness detection, Figure 25 validates the training and testing times of the traditional and proposed classification algorithms using the kidney disease dataset. The findings show that both training and testing activities take less time when using the suggested BOLD framework.

**Figure 26:**
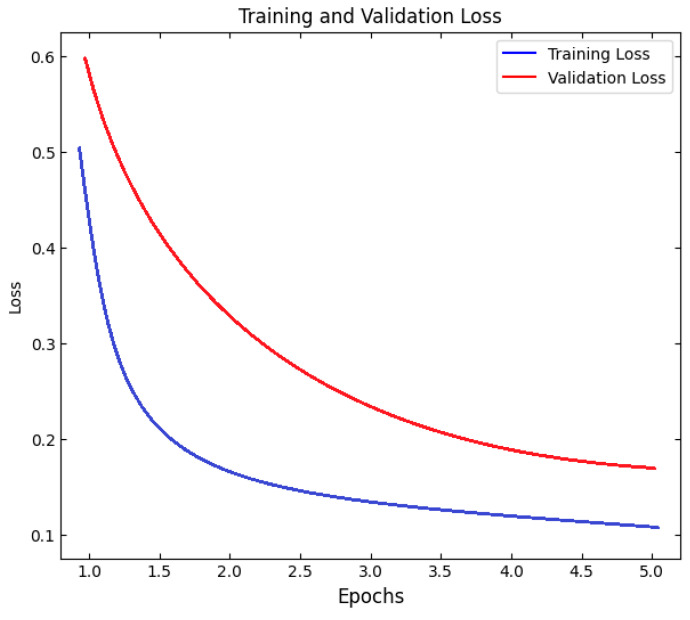
Loss value.

The BOLD model’s training and validation loss curves spanning several epochs are shown in Figure 26. The training loss is represented by the blue line, which shows how well the model performed on the training set. The validation loss is represented by the red line, which shows how well the model generalized on the validation set that had not yet been seen. The epoch-wise trend of loss reduction is illustrated by the curved, smooth lines; lower values signify better model convergence and performance.

Table 6 shows a thorough analysis of the total time required by several diabetes detection methods, emphasizing the computing demands and efficiency of each model. Despite its simplicity, the Logistic Regression (LR) model is an efficient linear model that can be quickly analyzed. It executes in 4.4 s. Known for its simple and obvious model structure, Decision Trees (DT) take 5.9 s to process since sorting and branching operations must be performed during training. The robust Support Vector Machine (SVM) technique for identifying the best hyper-plane to divide classes exhibits a longer computational time of 6.7 s, which is likely due to the extensive calculations required to determine margins and support vectors. Because it can analyze data in parallel, Random Forest (RF) stands out for having a comparatively faster execution time of 3.9 s. RF builds numerous decision trees and aggregates their findings for improved generalization. The computationally intense nature of K-Nearest Neighbors (KNN), another intensive method that computes the distances between all data points, is demonstrated by its completion time of 6.5 s. The proposed strategy, on the other hand, performs noticeably better than the conventional techniques and has an amazing execution time of just 0.8 s. This exceptional efficiency is attained by combining deep learning and powerful optimization approaches, which simplify the feature selection, preprocessing, and classification procedures and provide a very reliable and quick diabetes detection solution. The comparative analysis highlights the noteworthy benefits of the suggested approach concerning speed and computational effectiveness, rendering it an exceptional option for large-scale healthcare applications.

With an execution duration of only 0.8 s, the BOLD model demonstrates a considerable reduction in computational time when compared to other methods like Logistic Regression (4.4 s) and Support Vector Machine (6.7 s). It is appropriate for large-scale datasets and real-time applications due to its increased efficiency. The BOLD technique combines deep learning, optimization algorithms, and hybrid modelling (HO-RNN-LSTM) to produce a synergistic combination of accuracy, resilience, and flexibility.

## 5. Discussion

The paper’s original contribution is the design and development of a new automated diagnosis tool for the detection and classification of diabetes from the patients’ data. For accomplishing this objective, a novel AI methodology has been applied in this study. Here, the data mining operations, including data cleaning, normalization, feature optimization, and classification have been mainly applied for disease diagnosis. However, the complexity of such techniques needs to be analyzed in terms of cost and time. Also, it is required to compare the convergence analysis for determining the efficiency of the optimization techniques. These are the major limitations of the proposed study, which can be focused on by the authors in the next work. In addition, we planned to collect some real time data for the prediction of diabetes according to the medical history of patients. Moreover, cloud-integrated IoMT technology can be used further for the analysis and diagnosis of different chronic diseases.

Better feature selection, model training, and hyperparameter optimization are made possible by this integration, which improves SDN-IoT security and diabetes detection performance. The suggested method is thoroughly tested on a number of datasets, such as the kidney disease dataset, the Indonesia diabetes database, and the PIDD. This thorough evaluation guarantees the BOLD approach’s generalizability and efficacy across a range of healthcare circumstances and data.

Even though the BOLD technique offers notable efficiency gains, putting it into practice could necessitate a large investment in computing power, particularly when it comes to training deep learning models and doing hyperparameter optimization. In order to overcome this constraint, methods like model compression, distributed computation, or hardware acceleration may be investigated in order to lower resource requirements. The quality and quantity of data that is provided determines how well the BOLD approach performs, as is the case with many machine learning techniques. The model’s capacity to effectively generalize may be restricted by incomplete or biased datasets, which may also induce biases or mistakes in predictions. To address these issues, future research might concentrate on domain adaptation strategies or data augmentation approaches. Deep learning models are frequently criticized for being difficult to understand and interpret, especially those with sophisticated architectures like RNN-LSTM. Even if the BOLD approach places a higher priority on performance, efforts to improve the interpretability of the model using methods like feature importance analysis, attention mechanisms, or model distillation could increase user confidence and make it easier for domain experts to grasp the model.

The proposed BOLD framework potentially has a potent diagnostic capability. The authors, however, acknowledged in their paper that the framework might be limited in its applicability in various different environments and also its performance might decline over time due to several shortcomings. Firstly, the model’s dependence on multiple optimization-driven components like BPO for feature selection and DHO for hyperparameter tuning to a large extent increases the computational complexity. Therefore, the model’s performance might be limited when it is running on low-power or resource-constrained healthcare devices. The framework could potentially encounter difficulties in scaling to very large, high-dimensional medical databases, even though it obtains nice results for the datasets utilized in the experiments. In such a circumstance, it might be necessary to change the architecture further, incorporate parallel processing or distributed computing to be able to provide the response in real time. Conversely, the model’s restriction can also be the disparities in healthcare data from different regions and demographics. Anyway, the BOLD system remains quite versatile, and the authors can further enhance its adaptability by employing transfer learning, incremental learning, and domain adaptation techniques. This will enable the framework to extend its prediction capability to various chronic disease datasets, different scenarios of remote monitoring, and heterogeneous healthcare environments as long as suitable model updates and data integration are carried out.

## 6. Conclusions and Future Scope

This paper develops a new framework, BOLD, for the remote healthcare application system, where the combination of mining operations is performed to make an effective prediction from the patient data. The computation process starts with the collection of the PIDD dataset, which is a well-known and widely used diabetes database. After preprocessing the input dataset, several operations are carried out, such as cleaning, normalization, and dividing the training and testing set. Preprocessing aims to organize unprocessed data so that it will be easier to use and more beneficial for following processing steps. The preprocessed data is subsequently reduced in dimension by using the cutting-edge BPO technique to select the best features. The HO-RNN-LSTM can distinguish the diabetic and non-diabetic patients with high accuracy by using the selected training features. Also, it can modify the cell state by either eliminating or adding information, which is precisely controlled via gates. The RNN-LSTM hyper parameters are tuned using the DHO technique, which helps the classifier make accurate decisions with little computational effort. To compare and validate the outcomes of the suggested BOLD framework, a performance evaluation of its performance was conducted. The suggested model contains a number of mining processes, including cleaning, normalization, splitting, attribute selection, hyperparameter tweaking, and prediction, that aid in improving detection performance. Furthermore, the results are far better than those of the preceding models in terms of 99% accuracy, 99% precision, and a 2% miss rate. The current work can be improved in the future by creating a new framework that can manage many ailments like heart disease, diabetes, stroke, and other conditions for remote healthcare applications.

## Figures and Tables

**Table 1:** Survey on existing diabetes prediction models.

Ref.	Methods	Dataset Used	Pros and Cons
[33]	Tree based ensemble model	PIDD	Inaccurate prediction, and not highly reliable.
[34]	LSTM based decision support system	PIDD	Improved dataset training, and better prediction.
[35]	Ensemble of machine learning technique (GB, NB and LR)	PIDD	Effective dataset handling, high processing time, and low accuracy.
[36]	NB, RF, J48, and DT	Kidney disease dataset	High error rate, and lack of efficiency.
[37]	Cross validation model for type 2 diabetes prediction	PIDD	Optimized performance rate, high computational complexity, and increased feature dimensionality.
[38]	Heterogeneous ensemble classification model	PIDD	Ability to handle multi-type datasets with better efficiency, and better training performance.
[39]	Binary logistic regression model	PIDD	Increased recognition rate, and fast processing.
[40]	K-Means clustering with tree-based classification	Indonesia diabetic database	Increased time for training and testing operations, and ineffective prediction process.

**Table 2:** List of symbols and descriptions.

Variable	Description
$g_{i,j}$	Population set
m,n	Population member and problem variable
f	Random value
$U_{j}$	Upper bound
$L_{j}$	Lower bound
G	Matrix
Z	Objective function
$g_{i,j}^{Y_{1}}$	New status of pelican at exploration
$g_{i}^{Y_{2}}$	New status of pelican at exploitation
C	Constant value
itr	Current iteration
max_itr	Maximum number of iterations
$l_{j}$	Location of ith pelican
s	Random number between 0 to 1

**Table 3:** Statistical analysis of attributes.

Attributes	Mean	Standard Deviation	Minimum	Maximum
Pregnancies	3.8	3.4	0	16
Glucose	120.9	32	0	198
Blood pressure	69.1	19.4	0	121
Skin thickness	20.5	16	0	99
Insulin	79.8	115.2	0	848
BMI	31.9	7.9	0	67.4
Diabetes pedigree function	0.47	0.33	0.078	2.49
Age	33.2	11.8	21	83
Outcome (0 or 1)	-	-	0	1

**Table 4:** Performance study with recent deep learning algorithms.

Methods	Precision	Recall	Accuracy	AUC
CNN	0.756	0.772	0.781	0.81
CNN-LSTM	0.783	0.797	0.813	0.836
ConvLSTM	0.802	0.833	0.846	0.858
DCNN	0.819	0.842	0.862	0.912
SMOTE-Deep LSTM	0.954	0.946	0.996	0.983
Proposed	0.991	0.99	0.996	0.99

**Table 5:** Comparative study based on RMSE and kappa measures using Indonesia diabetic database.

Methods	RMSE	Kappa
CNN	0.476	0.671
CNN-LSTM	0.319	0.78
ConvLSTM	0.162	0.81
DCNN	0.078	0.89
SMOTE-Deep LSTM	0.027	0.94
Proposed	0.015	0.99

**Table 6:** Overall comparison based on time (s).

Methods	Overall Time (s)
LR	4.4 s
DT	5.9 s
SVM	6.7 s
RF	3.9 s
KNN	6.5 s
Proposed	0.8 s

## Data Availability

The datasets used and/or analyzed during the current study are available from the corresponding author on request.
